# Ultra-high dose rate radiotherapy overcomes radioresistance in head and neck squamous cell carcinoma

**DOI:** 10.1038/s41392-025-02184-0

**Published:** 2025-03-03

**Authors:** Hong-Shuai Li, Ruo Tang, Hua-Shan Shi, Zi-Jian Qin, Xiao-Yang Zhang, Yun-Fei Sun, Zhi-Gong Wei, Chao-Fan Ma, Liu Yang, Ye Chen, Zhe-Ran Liu, Li-Li Zhu, Wen Yang, Li Yang, Ai-Ning Xu, Zhuo Zhang, Shu-Qing Liao, Jin-Shui Shi, Jian-Jun Deng, Xiao-Zhong He, Xing-Chen Peng

**Affiliations:** 1 Department of Biotherapy, Cancer Center, and National Clinical Research Center for Geriatrics, West China Hospital of Sichuan University, Chengdu, China; 2 Sichuan Clinical Research Center of Biotherapy, Chengdu, China; 3 Institute of Fluid Physics, Chinese Academy of Engineering Physics, Mianyang, China; 4 Department of Applied Statistics, Social Science, and Humanities, New York University, New York, NY USA

**Keywords:** Cancer therapy, Cancer microenvironment, Tumour immunology

## Abstract

Radiotherapy (RT) resistance in head and neck squamous cell carcinoma (HNSCC) significantly hampers local control and patient prognosis. This study investigated the efficacy and molecular mechanisms of high-energy X-ray-based ultra-high dose rate radiotherapy (UHDR-RT) in overcoming RT resistance. The established RT-resistant HNSCC cell lines and animal models were subjected to UHDR-RT or conventional RT (Conv-RT) via a high-power rhodotron accelerator. Cellular assays assessed the malignant phenotype, viability, and degree of DNA damage, whereas in vivo evaluations focused on tumor proliferation and the tumor immune microenvironment (TiME). Transcriptome sequencing and Olink proteomics were employed to explore the underlying mechanisms involved. In vitro experiments indicated that UHDR-RT suppressed radioresistant cell proliferation and invasion, while promoting apoptosis and exacerbating DNA damage. In contrast, its efficacy in radiosensitive cells was comparable to that of Conv-RT. In vivo studies using patient-derived xenograft nude mice models demonstrated that UHDR-RT only partially reversed RT resistance. Transcriptomic and proteomic analyses of C57BL/6J mice models revealed the predominant role of TiME modulating in reversing radioresistance. Immunofluorescence and flow cytometry confirmed increased CD8^+^ T cells and an increased M1/M2 macrophage ratio post-UHDR-RT. Mechanistically, UHDR-RT activated CD8^+^ T cells, which stimulated M1 macrophages through paracrine IFN-γ signaling, thereby enhancing TiME activation. Furthermore, the activated M1 macrophages secreted CXCL9, which in turn reactivated CD8^+^ T cells, forming a feedforward loop that amplified TiME activation. This study elucidates the dual role of UHDR-RT in directly inducing DNA damage and modulating the TiME, highlighting its potential in treating radioresistant HNSCC.

## Introduction

Head and neck squamous cell carcinoma (HNSCC) ranks as the sixth most prevalent cancer worldwide, accounting for ~900,000 new cases and over 450,000 deaths annually, with mortality rates remaining stubbornly high despite advances in multimodal therapies.^1,2^ As a noninvasive and function-preserving therapy, radiotherapy (RT) serves as the cornerstone of curative-intent management for both early-stage and locally advanced HNSCC.^3^ However, due to the aggressive biological behavior of the disease, more than half (50–60%) of HNSCC patients undergoing RT experience local control failure and progress to recurrent or metastatic disease.^3–5^ Salvage therapies for recurrent or metastatic HNSCC, including re-irradiation, are limited by cumulative toxicity to critical structures (e.g., carotid arteries, brainstem, and spinal cord), which frequently causes severe complications such as osteoradionecrosis, dysphagia, and neurological deficits, ultimately diminishing quality of life and survival outcomes for this patient subset.^3–5^ The mechanisms underlying RT resistance in HNSCC are multifactorial and interconnected. Radiation-induced DNA damage repair, mediated by homologous recombination and nonhomologous end joining, enables tumor cells to survive lethal double-strand breaks.^4^ Concurrently, the hypoxic tumor microenvironment, a hallmark of HNSCC, activates hypoxia-inducible factor 1α-driven pathways that promote angiogenesis, metabolic reprogramming, and immune evasion.^6^ Cancer stem cells, which exhibit enhanced DNA repair capacity and resistance to apoptosis, further contribute to radioresistance and disease recurrence.^7^ Emerging evidence also implicates exosome-mediated transfer of oncogenic miRNAs and metabolic adaptations, such as enhanced glycolysis and glutamine dependency, in sustaining tumor survival post-RT.^8^ Alterations in the tumor immune microenvironment (TiME), cell cycle arrest, evasion of apoptosis, regulation by exosomes and noncoding RNAs, and anomalies in ferroptosis also play crucial roles in radioresistance.^4^ Currently, pharmacological treatment strategies include the use of radiosensitizers such as cisplatin, cetuximab, immune checkpoint inhibitors, HfO2, and inhibitors of apoptosis-related proteins.^9,10^ However, there are currently very few established and effective strategies for clinically reversing RT resistance, suggesting a critical unmet clinical need.^11,12^

From the perspective of RT technology development, over the past few decades, innovations in clinical RT have primarily focused on optimizing the physical aspects, with key breakthroughs centered around the precision of dose distribution and the modulation of radiation source energy.^13^ Currently, these technological advancements have limited efficacy against radioresistant tumors. Conventional radiotherapy (Conv-RT) techniques have reached the theoretical limits of physical optimization, with diminishing returns from further hardware improvements in breaking through the efficacy bottleneck. This suggests that relying solely on the optimization of physical parameters may not be an effective path to overcome radioresistance, highlighting the urgent need to shift toward biologically-oriented innovative RT modalities. Ultra-high dose rate radiotherapy (UHDR-RT) can deliver radiation doses at ultra-high dose rates (≥40 Gy/s) within a few pulses (on the millisecond scale) and has been shown to differentially alter the response of normal tissues and tumors to radiation.^13–15^ Therefore, UHDR-RT not only has a significantly faster irradiation completion speed but also allows higher irradiation doses to be applied to tumors under the same tissue-protecting conditions as Conv-RT (0.5–20 Gy/min).^13^ This approach holds the promise of overcoming RT resistance while greatly reducing the number of irradiation cycles required for Conv-RT and the associated manpower and material costs in a clinical setting. Moreover, it reduces damage to normal organs in patients, alleviates patient suffering, improves quality of life, and resolves issues related to organ motion during RT, achieving precision RT aims.^6^ Thus, UHDR-RT represents a highly promising and potentially transformative radiotherapy technique. In the context of radioresistance, Leavitt et al. demonstrated through murine models of multiple tumor types (including HNSCC) that electron UHDR-RT can overcome hypoxia-mediated tumor radioresistance.^16^ This study artificially induced acute hypoxia via physical clamping of blood vessels, which, albeit unable to replicate the chronic hypoxic conditions and immunosuppressive tumor microenvironment observed in actual tumors, revealed the potential of UHDR-RT to reverse radioresistance.

High-energy X-rays (HEXs) are the most widely used beam type in clinical RT settings, with deep penetration, low divergence, and affordability, making them highly anticipated for tumor control. However, owing to limitations in conversion efficiency and technical challenges, progress in the development of HEX-based UHDR-RT has been significantly slow.^6,13^ Gao et al. first reported the protective effect of HEX-based UHDR-RT on normal tissues, representing a breakthrough in radiation physics research.^6^ Unlike the technological pathway of linear accelerators, our team developed a HEX-based high-power rhodotron accelerator capable of achieving a high dose rate of 100 Gy/s at a distance of 0.8 m from the target, with characteristics of a high duty cycle, high efficiency, high beam power, and high beam quality.^17^ This accelerator can repeatedly accelerate electrons in the same acceleration cavity to near-light speed, efficiently producing high-power, high-energy electron beams that conduct the UHDR-RT while also meeting the requirements for multiple irradiation angles.^17^ Despite these advancements, no studies have yet investigated HEX-based UHDR-RT in reversing HNSCC radioresistance.

Therefore, in this study, we aimed to determine whether HEX-based UHDR-RT can reverse RT resistance and elucidate the underlying molecular mechanisms utilized by the rhodotron accelerator.

## Results

### Rhodotron accelerator shows good performance and beam output quality

The rhodotron accelerator of the CAEP was employed to conduct both UHDR-RT and Conv-RT, the schematic diagram of which is shown in supplementary Fig. 1a. The layout of the rhodotron accelerator and irradiation platform is illustrated in supplementary Fig. 1b. The electron beam was directed through a stainless steel target. Three lead bricks were used as collimators to form the X-ray field. A PMMA holder was used to support a mouse or petri dish. The holder was mechanically aligned to the platform with a positional error of less than 1 mm throughout the experiments. An EBT4 film was placed between the sample and the holder. The TW30013 ionization chamber (PTW farmer type) and electrometer (PTW Unidos Romeo) were mounted on the centerline to allow immediate monitoring of the irradiation process. The final absolute dose for each experiment is confirmed by the 24-h film measurement. In our experiments, the average dose rate was changed to adjust the time structure of the irradiation. For UHDR-RT, the peak dose rate was 176 Gy/s, and the average dose rate was 88 Gy/s for the duration of irradiation (Supplementary Fig. 1c). For Conv-RT, the peak dose rate was 176 Gy/s, and the average dose rate was ~3.095 Gy/min (Supplementary Fig. 1d). Supplementary Fig. 1e, f shows the dose distribution in the irradiated area for the cell experiments, and Supplementary Fig. 1g, h shows those for the animal study. The EBT4 film demonstrated accurate dose administration and good dose distribution during cell (Supplementary Fig. 1i, j) and animal experiments (Supplementary Fig. 1k, l), with the specific parameters listed in Supplementary Table 1.

### UHDR-RT significantly inhibits the malignant phenotype and increases the degree of DNA damage of RT-resistant cell lines

We successfully constructed and validated the radioresistant human tongue SCC cell line CAL33_R and the mouse oral SCC cell line MOC1_R. The CCK-8 assay (Supplementary Fig. 2a, b) and colony formation assay (Supplementary Fig. 2c–f) were used to validate the radioresistant cell lines, and radiation doses of 6 Gy for CAL33_R and 4 Gy for MOC1_R were used for subsequent cell and animal experiments, respectively.

**Fig. 1:**
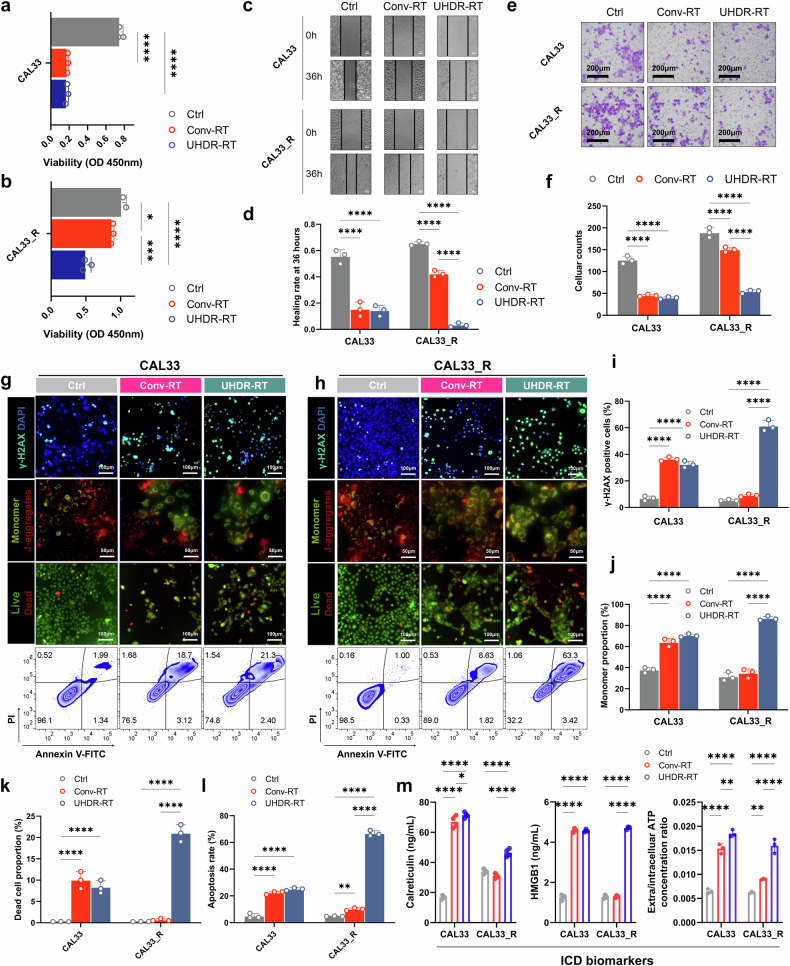
Cellular experiments demonstrated that UHDR-RT reversed radioresistance and led to increased DNA damage. Compared with Conv-RT, UHDR-RT significantly inhibited CAL33_R cell proliferation (**a**, **b**), migration (**c**, **d**), and infiltration ability (**e**, **f**), increased DNA damage (**g**–**i**), decreased the mitochondrial membrane potential (**g**, **h**, **j**), and increased the degree of cell death (**g**, **h**, **k**), apoptosis (**g**, **h**, **l**), and ICD (**m**). Ctrl control; Conv-RT conventional radiotherapy; UHDR-RT ultra-high dose rate radiotherapy; ICD immunogenic cell death. *p < 0.05; **p < 0.01; ***p < 0.001; ****p < 0.0001. The data are presented as the mean ± SEM (**a**, **b**, **d**, **f**, and **i**–**m**). Comparisons were performed using one-way ANOVA with Tukey’s test for multiple comparisons

First, in the RT-sensitive treatment group, we found that UHDR-RT exhibited comparable tumor control ability to Conv-RT, including in terms of proliferation, infiltration, migration, apoptosis, and DNA damage (Fig. 1). However, the CCK-8 results revealed that UHDR-RT markedly reduced the cell viability by 30% of CAL33_R cells compared with Conv-RT (Fig. 1a, b). In addition, compared with Conv-RT, the scratch wound healing assay (Fig. 1c, d) and transwell infiltration assay (Fig. 1e, f) suggested that UHDR-RT significantly suppressed the migration and invasion ability of CAL33_R cells. Furthermore, we observed that UHDR-RT significantly increased the degree of cellular DNA damage by nearly 50% (more γ-H2AX-positive cells) compared with Conv-RT in CAL33_R cells (Fig. 1g–i) while reducing mitochondrial membrane potential levels (more monomers) by approximately twofold (Fig. 1g, h, j). Correspondingly, we also observed more dead cells (Fig. 1g, h, k), which was validated by the FCM apoptosis assay (Fig. 1g, h, l). The above findings suggested that UHDR-RT significantly reversed radiation resistance compared with Conv-RT with the same radiation dose in the RT-resistant background. Previous studies have reported that DNA damage, as a kind of damage-associated molecular patterns, can induce immunogenic cell death (ICD).^18^ Therefore, we also assessed ICD-related markers including calreticulin, HMGB1, and ATP levels (Fig. 1m) (Supplementary Fig. 3a). The results showed that consistent with the above findings, UHDR-RT significantly increased the level of ICD markers in CAL33_R. Moreover, we also conducted FCM analysis on the reactive oxygen species (ROS) levels, and the results revealed that UHDR-RT significantly reduced the ROS levels compared with Conv-RT, both in CAL33 and CAL33_R cells (Supplementary Fig. 3b, c). The reduction in ROS in the UHDR-RT subgroup suggests that UHDR-RT reverses RT resistance primarily through direct DNA damage rather than indirect cytotoxicity mediated by ROS.

### UHDR-RT reverses RT resistance in mouse models by activating TiME and increasing the DNA damage level

**Fig. 2:**
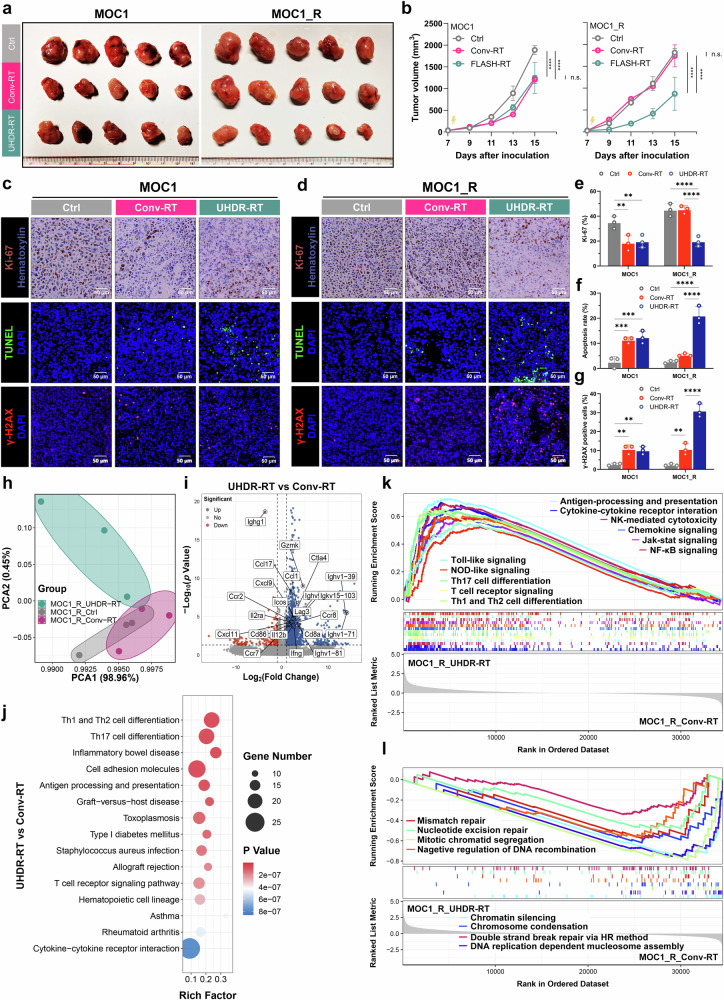
Mouse experiments confirmed that UHDR-RT reversed radioresistance and led to TiME activation and DNA damage. C57BL/6J mouse experiments confirmed that in the MOC1_R group, tumors (*n* = 5) in the UHDR-RT group achieved better tumor control than those in the Conv-RT group (*n* = 5) (**a**, **b**). Ki-67 staining, TUNEL staining, and DNA damage IF of mouse tumor tissues revealed that UHDR-RT significantly increased DNA damage and apoptosis levels and decreased tumor proliferative activity in the MOC1_R group (**c**–**g**). Transcriptome PCA revealed subgroups within the MOC1_R group (**h**), volcano plots revealed differential genes in the UHDR-RT and Conv-RT subgroups within the MOC1_R group (**i**), and KEGG analysis revealed an enriched signaling pathway for the UHDR-RT subgroup within the MOC1_R group (**j**). GSEA enrichment analysis revealed upregulated (**k**) and downregulated (**l**) pathways in the UHDR-RT subgroup. Ctrl control; Conv-RT conventional radiotherapy; UHDR-RT ultra-high dose rate radiotherapy; PCA principal component analysis. ns not significant; *p < 0.05; **p < 0.01; ***p < 0.001; ****p < 0.0001. The data are presented as the mean ± SEM (**b** and **e**–**g**). Comparisons were performed using two-way (**b**) or one-way (**e**–**g**) ANOVA with Tukey’s test for multiple comparisons

C57BL/6J mouse efficacy evaluation models were constructed by subcutaneously injecting MOC1 and MOC1_R cells to investigate whether UHDR-RT could reverse RT resistance in vivo. Consistent with the results of the cellular experiments, we found that in a non-RT-resistant context, UHDR-RT demonstrated a comparable tumor control effect to that of Conv-RT (Fig. 2). However, compared with Conv-RT, UHDR-RT reduced the tumor volume in the MOC1_R group by nearly twofold (Fig. 2a, b). Compared with those in the Conv-RT subgroup, Ki-67 staining, TUNEL staining, and DNA damage IF of mouse tumor tissues revealed that the UHDR-RT subgroup in the MOC1_R group presented markedly increased DNA damage (by threefold) and apoptosis levels (by fourfold) and decreased tumor proliferative activity (by twofold) (Fig. 2c–g). We subsequently performed transcriptome sequencing of mouse tumor tissues. Principal component analysis (PCA) results suggested that the UHDR-RT subgroup of MOC1_R was significantly distinguished from the other two subgroups (Fig. 2h). Compared with the Conv-RT subgroup, the UHDR-RT subgroup presented significantly upregulated expression of immune-related genes (including *Cd8a*, *Gzmk*, *Ifng*, *Ccl17*, *Cxcl9*, etc.) (Fig. 2i). KEGG enrichment analysis revealed that the UHDR-RT group was significantly enriched in pathways related to T-cell activation, including Th1/Th2 cell differentiation, antigen processing and presentation, the T-cell receptor signaling pathway, and cytokine‒cytokine receptor interactions (Fig. 2j). GSEA similarly suggested that UHDR-RT upregulated intrinsic and adaptive immunity-related pathways (Fig. 2k). In addition, we observed that UHDR-RT also significantly downregulated DNA damage repair-related pathways (Fig. 2l), which was consistent with the DNA damage staining results. In line with the ELISA results observed in the cell experiments, UHDR-RT also significantly increased the transcriptomic levels of ICD-related genes in MOC1_R tumors (Supplementary Fig. 4a), indicating the ICD induced by DNA damage. The above findings were supported by the results of WGCNA (Supplementary Fig. 4), which revealed that the genes in the UHDR-RT subset of the MOC1_R group were highly associated with the turquoise module compared with the other subgroups (*r* = 0.52, *p* < 1e-200). The KEGG enrichment analysis of genes within the module revealed significant enrichment of pathways associated with both intrinsic and adaptive immune responses (Supplementary Fig. 4b–g). The experimental results confirmed the possible mechanisms of UHDR-RT in reversing RT resistance.

**Fig. 3:**
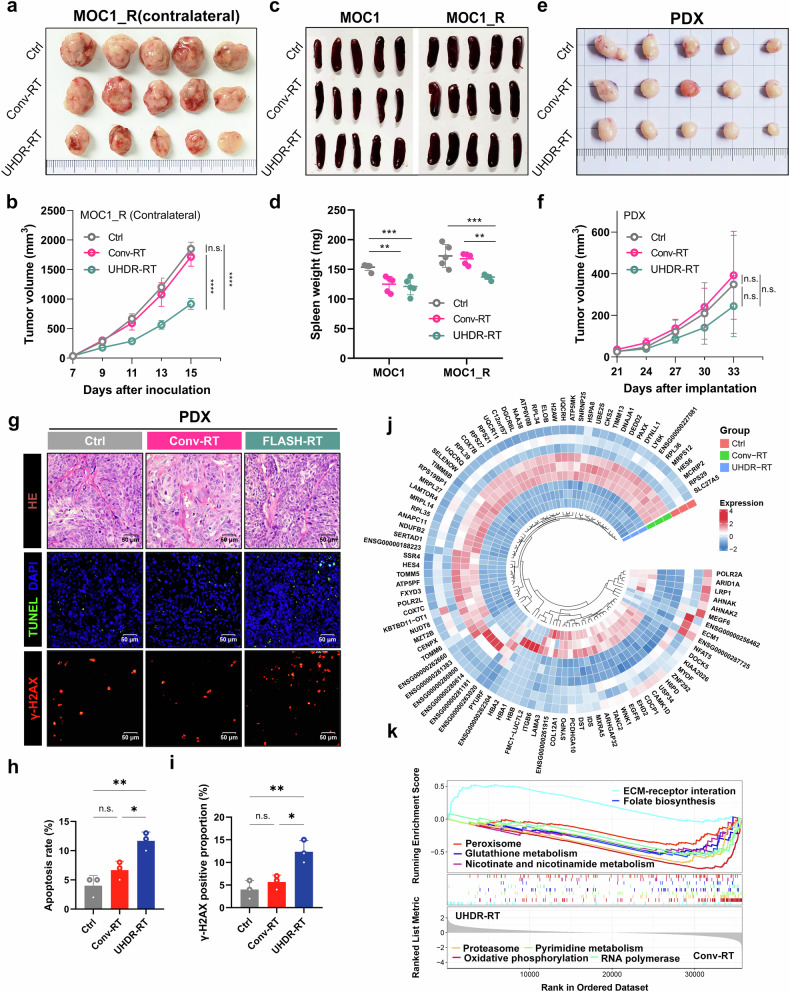
Abscopal effects and PDX experiments confirmed the crucial role of TiME activation in reversing resistance. Compared with Conv-RT (*n* = 5), UHDR-RT (*n* = 5) also significantly decreased the volume of contralateral tumors (**a**, **b**). Compared with Conv-RT, UHDR-RT significantly reduced the spleen burden in the MOC1_R group (**c, d**). Compared with Conv-RT, UHDR-RT (*n* = 5) reduced the PDX volume (*n* = 5) but not significantly (**e**, **f**). HE staining revealed typical squamous carcinoma histologic features, and TUNEL staining and DNA damage staining suggested that UHDR-RT significantly increased apoptosis and DNA damage levels (**g**–**i**). The ring heat plot shows differential gene expression profiles between groups (**j**), and GSEA demonstrated different signaling pathway enrichment between the two groups (**k**). Ctrl control; Conv-RT conventional radiotherapy; UHDR-RT ultra-high dose rate radiotherapy; PDX patient-derived xenograft; HE staining hematoxylin‒eosin staining; TiME tumor immune microenvironment; ns not significant; *p < 0.05; **p < 0.01; ***p < 0.001; ****p < 0.0001. The data are presented as the mean ± SEM (**b**, **d**, **f**, and **h**, **i**). Comparisons were performed using two-way (**b**, **f**) or one-way (**d**, **h**, **i**) ANOVA with Tukey’s test for multiple comparisons

Furthermore, to clarify which immune-related killing and direct DNA damage play major roles in the reversal of RT resistance, we observed the abscopal effects on nontarget tumors. In addition to the target tumors, UHDR-RT significantly reduced the nontarget tumor volume in the MOC1_R group compared with that in the Conv-RT group (Fig. 3a, b). Similarly, spleen weight measurements suggested that UHDR-RT significantly reduced the spleen burden of the mice in the MOC1_R group (Fig. 3c, d). To exclude the possibility that the observed effects on DNA damage were due to immune-related killing, we compared the efficacy of UHDR-RT and Conv-RT in the RT-resistant laryngeal SCC PDX model (construction and identification are shown in supplementary Fig 5), and the results suggested that UHDR-RT mildly, but not significantly, reduced tumor volume (Fig. 3e, f). Similarly, TUNEL fluorescence staining did not reveal a killing advantage of UHDR-RT over Conv-RT, but UHDR-RT increased the apoptosis rate compared with that of the control group by 5% (Fig. 3g, h). In addition, DNA damage (γ-H2AX) fluorescence staining suggested that UHDR-RT *notably* increased the level of DNA damage by 2-fold in tumor tissues compared with Conv-RT (Fig. 3g, i). A ring heatmap revealed significantly different differential gene expression profiles between the different groups (Fig. 3j). In addition, GSEA enrichment analysis revealed that, compared with Conv-RT, UHDR-RT significantly downregulated numerous pathways associated with DNA damage repair (mainly oxidative phosphorylation; RNA polymerase, peroxisome, proteasome, and folate biosynthesis; glutathione metabolism; nicotinate and nicotinamide metabolism; and pyrimidine metabolism pathways) (Fig. 3k). These results suggest that UHDR-RT can significantly inhibit pathways related to DNA damage repair in the RT-resistant PDX model, which in turn increases DNA damage and apoptosis. Despite its theoretical promise, adequate tumor control was not achieved in the RT-resistant laryngeal SCC PDX model.

**Fig. 4:**
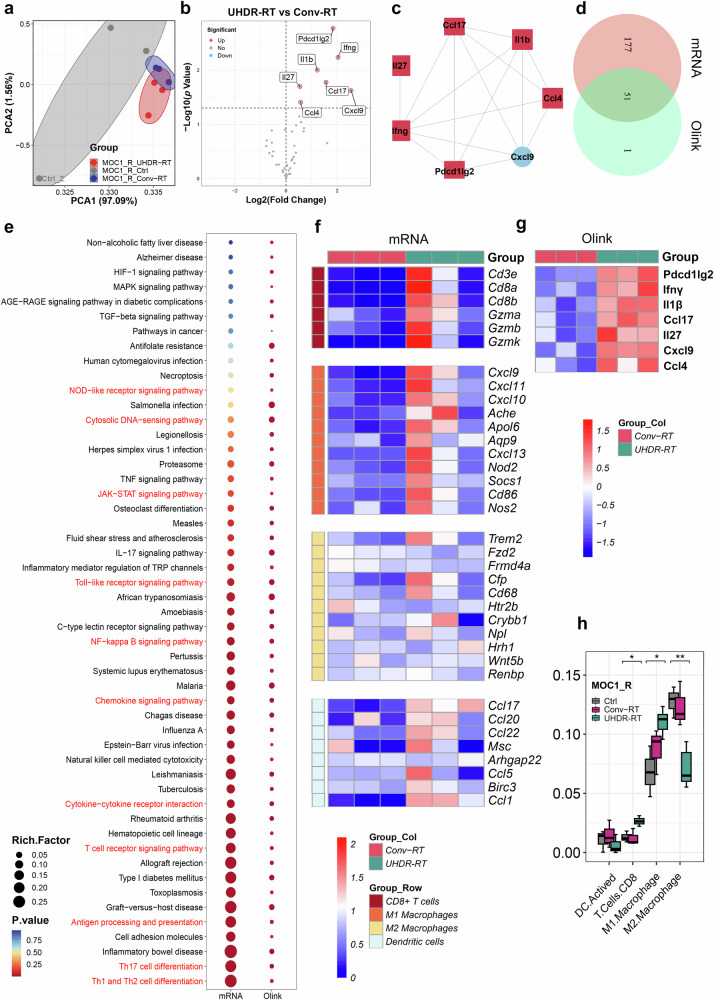
Integrative analysis of transcriptomics and proteomics confirmed the activation of the TiME. Olink proteome PCA revealed subgroup clustering of the MOC1_R group (**a**). Volcano plot showing significantly upregulated proteins in the UHDR-RT subgroup compared with the Conv-RT subgroup (**b**) and an interaction between these proteins (**c**). There is a cross-relationship between pathways enriched in proteome-differentiated proteins and pathways enriched in transcriptome-differentiated genes, and the cross-pathways (**d**) are strongly associated with intrinsic as well as adaptive immune responses (**e**). Transcriptomic data (**f**), and Olink data (**g**) revealed significant upregulation of CD8^+^ T-cell and M1-type macrophage surface markers in the UHDR-RT subgroup. Cybersort analysis revealed differences in TiME infiltration between the UHDR-RT subgroup and the Conv-RT subgroup (**h**). Ctrl control; Conv-RT conventional radiotherapy; UHDR-RT ultra-high dose rate radiotherapy; TiME tumor immune microenvironment; DEGs differentially expressed genes; DEPs differentially expressed proteins; IHC immunohistochemistry; IF immunofluorescence. *p < 0.05; **p < 0.01; ***p < 0.001; ****p < 0.0001. The data are presented as the median and interquartile range (**h**). The boxplots indicate median (center), 25th and 75th percentiles (bounds of box), and minimum and maximum (whiskers). Comparisons were performed using one-way ANOVA method

We simultaneously performed Olink proteomic analysis (the panel is shown in Supplementary Table 2 and results in Supplementary Table 3) on tumor tissues, and the PCA subgroups showed results consistent with those of transcriptome sequencing (Fig. 4a). The volcano plot revealed seven interconnected proteins (including PDCD1LG2, IFN-γ, IL-1β, CCL17, IL-27, CXCL9, and CCL4) that were significantly upregulated in the MOC1_R group (Fig. 4b, c). Enrichment analysis revealed 51 cross-shared pathways between the proteome and transcriptome, with significant enrichment of immune-related pathways (Fig. 4d, e). The heatmap of immune cell markers revealed that CD8^+^ T cells and M1-type macrophages differed significantly between the groups (Fig. 4f, g). To determine which immune system cell type might be involved in the immune response, we performed a Cybersort immune infiltration analysis.^19,20^ The results suggested that in the MOC1_R group, UHDR-RT significantly increased the proportions of CD8^+^ T cells and M1-type macrophages and significantly decreased the proportion of M2-type macrophages (Fig. 4h).

Not surprisingly, the control group in the MOC1 group was significantly different from the other two groups (Supplementary Fig. 6a). Compared with the Conv-RT subgroup, the UHDR-RT subgroup did not upregulate the expression of immune-related genes (Supplementary Fig. 6b). Consistently, KEGG enrichment analysis and GSEA did not reveal enrichment of adaptive immune activating pathways (supplementary Fig. 6c, d). Cybersort analysis revealed no significant difference in immune cell infiltration between the UHDR-RT group and the Conv-RT group (Supplementary Fig. 6e, f). Proteomic analysis revealed similar results (Supplementary Fig. 6g, h). Combined with the above findings, we tentatively speculated that UHDR-RT may promote the conversion of M2-type macrophages to M1-type macrophages, which in turn secrete chemokines to stimulate the infiltration of CD8^+^ T cells and ultimately reverse RT resistance.

### CD8^+^ T cell activation accompanied by macrophage polarization leads to TiME activation

**Fig. 5:**
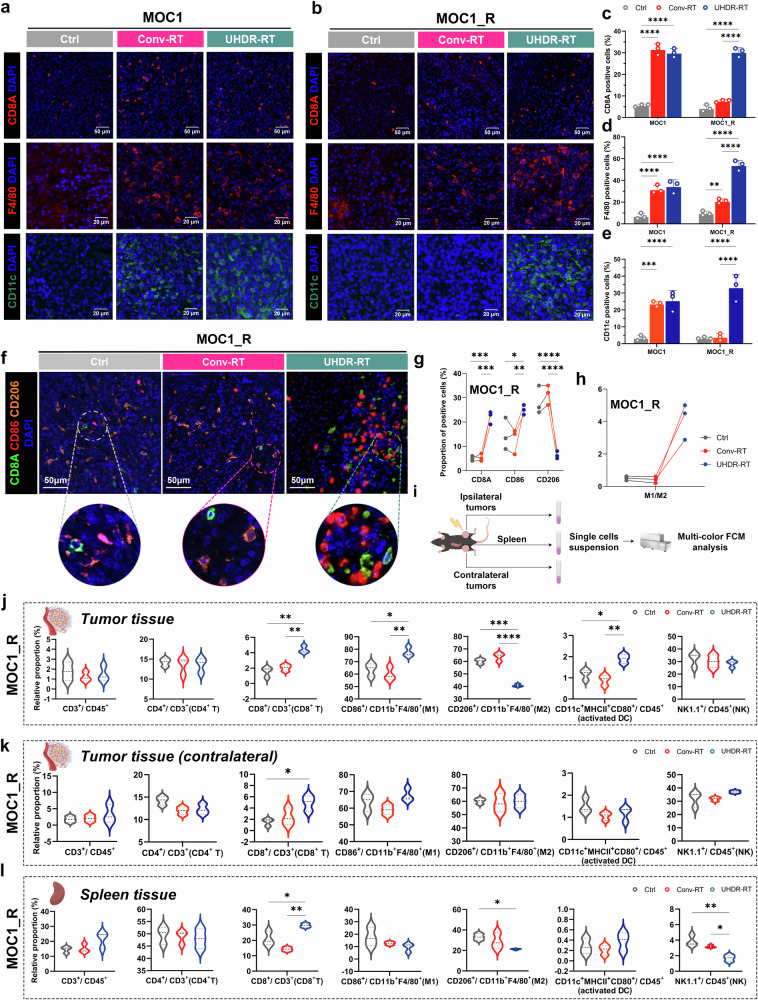
Significant aggregation of CD8^+^ T cells along with notable M1 polarization of macrophages led to TiME activation. A monochromatic IF assay suggested significant aggregation of CD8^+^ T cells, macrophages, and DCs in the UHDR-RT subgroup of the MOC1_R group (**a**–**e**), and multicolor IF verified the colocalization of CD8^+^ T cells and macrophages as well as significant M1 polarization of macrophages (**f**–**h**). The multi-color FCM scheme (**i**). The results of tumor and spleen multicolor FCM analysis were consistent with those of IF (**j**–**l**). Ctrl control; Conv-RT conventional radiotherapy; UHDR-RT ultra-high dose rate radiotherapy; DC dendritic cells; TiME tumor immune microenvironment. *p < 0.05; **p < 0.01; ***p < 0.001; ****p < 0.0001. The data are presented as the mean ± SEM (**c**–**e**) or median and interquartile range (**j–l**). Comparisons were performed using one-way (**c**–**e**) ANOVA with Tukey’s test for multiple comparisons

Consistent with the results of the transcriptome sequencing analysis, significant changes in CD8^+^ T cells and macrophages in the TiME were observed via IF (Fig. 5a–d). We also performed IF assays for DCs (Fig. 5a, b, e), Tregs, and neutrophils (supplementary Fig. 7a-d), but significant differences were only found in the proportion of DCs between UHDR-RT and Conv-RT. To clarify the effects of UHDR-RT on the colocalization of CD8^+^ T cells with macrophages and macrophage polarization status in the MOC1_R group, multicolor IF staining was conducted (Fig. 5f–h), and the results suggested that UHDR-RT significantly increased the infiltration of CD8^+^ T cells in the TiME by nearly 20%, accompanied by a significant increase in M1-type macrophages by 10% and a significant decrease in M2-type macrophages by more than 20%. In addition, to clarify the specific effects of UHDR-RT on immune cell distribution in the TiME, we also performed multicolor FCM analysis (Fig. 5i–l). The specific gating strategies are shown in Supplementary Fig. 8. Consistent with the results of multicolor IF, the results of both tumor tissue and spleen analyses suggested that UHDR-RT increased the proportion of CD8^+^ T cells accompanied by macrophage polarization, although the increase of M1 macrophages in the spleen was not significant. Taken together, these findings suggest that UHDR-RT reverses RT resistance by activating CD8^+^ T cells and promoting M1 polarization of macrophages in the suppressive TiME. Another noteworthy point is that, compared to the Conv-RT group, the UHDR-irradiated tumors exhibited significantly higher levels of activated dendritic cells on the irradiated side; however, no significant increase was observed on the non-irradiated side. This indirectly confirms that DNA damage induced by direct UHDR irradiation plays a crucial role in activating dendritic cells (namely ICD), which is important for initiating subsequent adaptive immune responses.

**Fig. 6:**
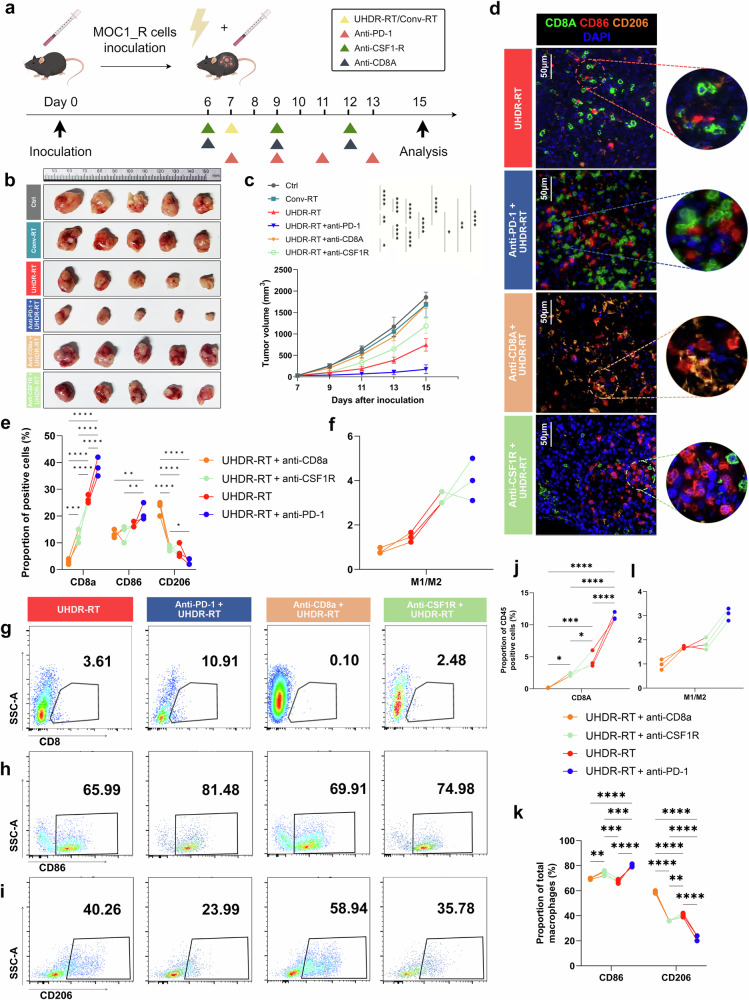
Endogenous antibody blockade experiments in mice confirmed the crucial role of CD8^+^ T cells and macrophages. Subcutaneous injection of the MOC1_R cell line was used to construct a radiotherapy-resistant C57BL/6J mouse model, which received Conv-RT, UHDR-RT, or UHDR-RT combined with anti-PD-1, anti-CD8a or anti-CSF1R (**a**, **b**). Tumor growth curves of the different groups (**c**). Multicolor immunofluorescence demonstrated the colocalization of CD8^+^ T cells and macrophages in response to different treatments (**d**–**f**). FCM was used to determine the proportions of CD8^+^ T cells (**g**) and macrophages (**h**, **i**) in the different treatment groups. The proportions of CD8-, CD86- and CD206-positive cells (**j**, **k**) were calculated, and the M1/M2 ratio was computed (**l**). Ctrl control; Conv-RT conventional radiotherapy; UHDR-RT ultra-high dose rate radiotherapy. *p < 0.05; **p < 0.01; ***p < 0.001; ****p < 0.0001. The data are presented as the mean ± SEM (**c**). Comparisons were performed using two-way (**c**) or one-way (**e**, **j**, **k**) ANOVA with the Tukey’s test for multiple comparisons

To further validate the essential role of CD8^+^ T cells and macrophages in overcoming RT resistance, we conducted endogenous cell blockade assays in a C57BL/6J mouse model. We employed PD-1 monoclonal antibodies to activate CD8^+^ T cells and CD8A monoclonal antibodies to block them. Additionally, CSF1R monoclonal antibodies were used to inhibit macrophages (Fig. 6a). The tumor growth curves demonstrated that tumors in the UHDR-RT group treated with the PD-1 monoclonal antibody exhibited significant shrinkage compared with those in the UHDR-RT group alone (Fig. 6b, c). In contrast, tumors significantly increased in size by nearly threefold after CD8^+^ T-cell blockade (Fig. 6b, c). These findings suggest that T-cell activation is crucial for the efficacy of UHDR-RT. Blocking macrophages also led to a noticeable enlargement of tumors by nearly onefold in the UHDR-RT group, although the effect was less pronounced than that of CD8^+^ T-cell blockade (Fig. 6b, c). These observations were corroborated by the Ki-67 and TUNEL staining results, despite the absence of significant differences in γ-H2AX expression among the groups (Supplementary Fig. 9a–d). These findings suggest the crucial role of CD8^+^ T cells and macrophages in reversing RT resistance, with CD8^+^ T cells playing a particularly significant role.

Subsequent multicolor IF assays confirmed these results (Fig. 6d–f). In the anti-PD-1 subgroup, CD8^+^ T cells and M1-type macrophages were significantly colocalized. However, in the anti-CD8A subgroup, M1-type macrophages were significantly reduced by nearly 10%, whereas M2-type macrophages were significantly increased by 20% compared with the anti-PD-1 subgroup. In the anti-CSF1R subgroup, notably fewer CD8^+^ T cells colocalized with M1-type macrophages. These findings suggest a reciprocal promotion relationship between CD8^+^ T cells and M1-type macrophages within the TiME activated by UHDR-RT. Furthermore, multicolor FCM analysis revealed that the proportion of CD8^+^ T cells in the anti-CD8A and anti-CSF1R subgroups was significantly lower than that in the UHDR-RT subgroup. In contrast, anti-PD-1 treatment further increased the proportion of CD8^+^ T cells compared with that in the UHDR-RT group (Fig. 6g, j). Moreover, the M1/M2 ratio in the anti-CD8A subgroup was significantly lower than that in the UHDR-RT subgroup (Fig. 6h-i, k-l). This finding further validated the reciprocal promotion between CD8^+^ T cells and macrophages.

### UHDR-RT can directly activate CD8^+^ T cells while promoting M1-type macrophage polarization

**Fig. 7:**
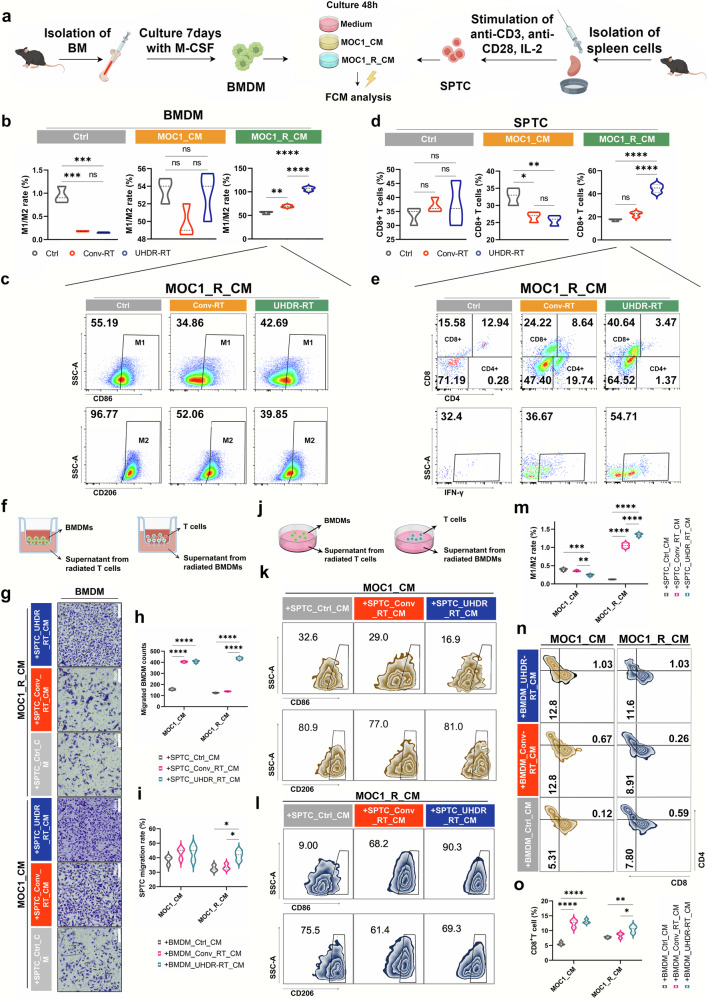
In vitro experiments demonstrated the interplay between CD8^+^ T cells and macrophages induced by UHDR-RT. Independent in vitro irradiation assay procedure (**a**). BMDMs (**b**, **c**) and SPTCs (**d**, **e**) that were cultured with different CM were treated with different radiotherapy modalities and then subjected to FCM. Transwell assay design (**f**). Transwell staining results of BMDMs (**g**). Statistical analysis of the transwell assay results for BMDMs (**h**). Statistical analysis of the transwell assay results for SPTCs (**i**). Co-culture assay design diagram (**j**). FCM results of the co-culture assay for BMDMs (**k**, **l**). Statistical analysis of the FCM results of the co-culture assay for BMDMs (**m**). FCM results of the co-culture assay for SPTC (**n**). Statistical analysis of the FCM results of the co-culture assay for SPTCs (**o**). Ctrl control; Conv-RT conventional radiotherapy; UHDR-RT ultra-high dose rate radiotherapy; BMDMs bone marrow-derived macrophages; SPTCs spleen-derived T cells; CM tumor-conditioned medium; FCM flow cytometry. *p < 0.05; **p < 0.01; ***p < 0.001; ****p < 0.0001. The data are presented as the median and interquartile range (**b**–**d**, **h**, **i**, **m**, **o**). Comparisons were performed using one-way ANOVA with Tukey’s test for multiple comparisons

To clarify the effects of UHDR-RT on T cells and macrophages, we first performed independent in vitro irradiation assays (Fig. 7a). First, we treated mouse-derived BMDMs with CM from MOC1 or MOC1_R for 48 h and then irradiated them with UHDR-RT or Conv-RT. FCM analysis was performed 24 h later. Compared with the Conv-RT subgroup, in the MOC1_R_CM subgroup, the UHDR-RT subgroup presented a significantly increased M1/M2 ratio, whereas there was no significant difference between the two subgroups within either the MOC1_CM or the Ctrl group (Fig. 7b, c). Notably, we also found that the higher M1/M2 ratio in the UHDR-RT group was caused mainly by a decrease in the proportion of M2-type macrophages rather than an increase in the proportion of M1-type macrophages. This finding suggests another source for the significantly increased number of M1-type macrophages observed in the preceding mouse experiments (Fig. 7c). Similarly, we also treated mouse spleen-derived T cells (SPTC) with CM from MOC1 and MOC1_R for 48 h, followed by UHDR-RT or Conv-RT irradiation, respectively. FCM analysis was performed 24 h later. The results suggested that in the MOC1_R_CM subgroup, the UHDR-RT subgroup significantly increased the proportion of CD8^+^ T cells by nearly 20% compared with the Conv-RT subgroup, whereas there was no significant difference between the two subgroups within both the MOC1_CM group and the Ctrl group (Fig. 7d, e). Notably, CD8^+^ T cells not only presented increased proportions but also increased activity (increased proportions of IFN-γ^+^ T cells) (Fig. 7e). The results of the above experiments suggest that UHDR-RT is able to activate CD8^+^ T cells directly while promoting the polarization of macrophages. This finding is consistent with the findings of previous mouse studies.

To clarify the interaction between T cells and macrophages, we performed the in vitro macrophage and T cell interaction assays. Firstly, MOC1_CM or MOC1_R_CM that had been cultured with irradiated BMDMs or SPTCs was collected. We then designed transwell migration assays of BMDMs with SPTC supernatants or SPTCs with BMDM supernatants (Fig. 7f). Transwell assays of BMDMs confirmed that MOC1_R_CM cultured with SPTCs (irradiated with UHDR-RT) significantly increased the migratory ability of BMDMs by 10% relative to other subgroups (Fig. 7g, h). We obtained similar results in the transwell assay of SPTCs (Fig. 7i). In addition, we design co-culture assays of BMDMs with SPTC supernatants or SPTCs with BMDM supernatants (Fig. 7j). The FCM results confirmed that MOC1_R_CM cultured with SPTCs (irradiated with UHDR-RT) significantly increased the proportion of M1-type macrophages in BMDMs relative to that in the other subgroups, accompanied by a significant increase in the M1/M2 ratio (Fig. 7k–m). Surprisingly, unlike the increase in the M1/M2 ratio in BMDMs resulting from UHDR-RT alone, SPTC supernatants increased this ratio primarily by increasing the number of M1-type macrophages, whereas the former increased this ratio primarily by decreasing the number of M2-type macrophages. These findings suggest that UHDR-RT increases the M1/M2 ratio, on the one hand, by directly reducing M2-type macrophages and, on the other hand, by increasing M1-type macrophages through the paracrine stimulation of T cells. In addition, we obtained similar results in the co-culture assay of SPTCs (Fig. 7n, o), where we observed that compared with other subgroups, MOC1_R_CM cultured with BMDMs (irradiated with UHDR-RT) significantly increased the proportion of CD8^+^ T cells, suggesting that macrophages are also capable of affecting CD8^+^ T cells in a paracrine manner.

### The paracrine-positive feedback loop between CD8^+^ T cells and macrophages induced by UHDR-RT

**Fig. 8:**
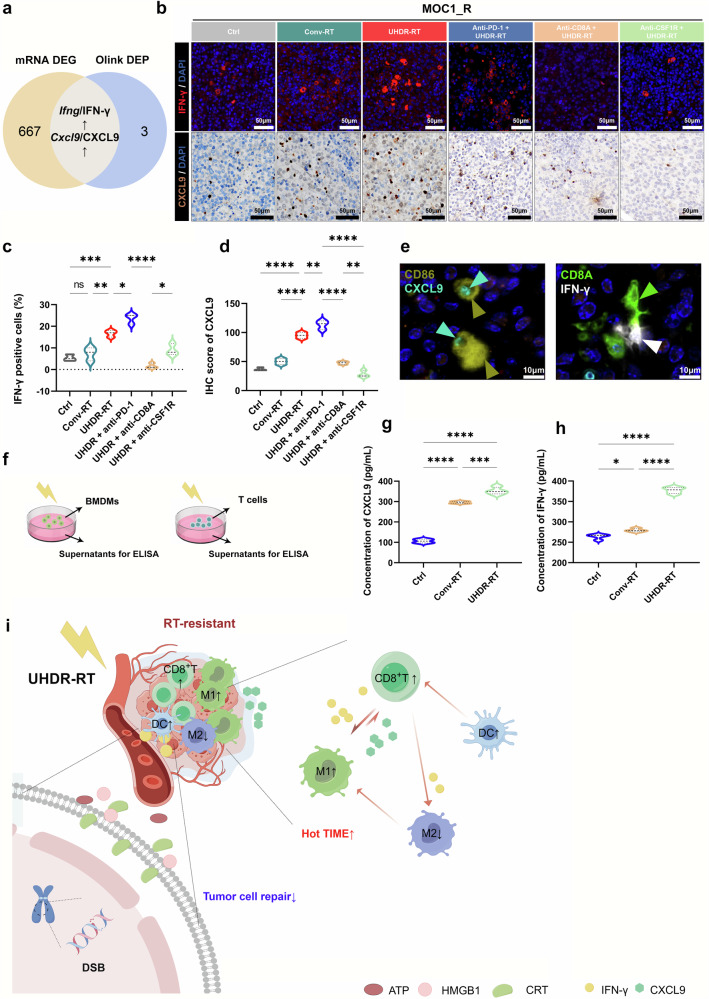
The paracrine positive feedback loop between CD8^+^ T cells and macrophages induced by UHDR-RT. Venn diagram showing the intersecting genes/proteins obtained from transcriptome sequencing and proteomics analysis (**a**). IFN-γ and CXCL9 IF and IHC validation results (**b**–**d**). IF confirmed the colocalization of M1 macrophages with CXCL9 and of CD8^+^ T cells with IFN-γ (**e**). ELISAs verified the significant upregulation of CXCL9 and IFN-γ in the supernatants of radiated BMDMs and SPTCs, respectively (**f**–**h**). Mechanism diagram of UHDR-RT overcoming radioresistance. (**i**). Ctrl control; Conv-RT conventional radiotherapy; UHDR-RT ultra-high dose rate radiotherapy; BMDM bone marrow-derived macrophage; SPTC spleen-derived T cell; IHC immunohistochemistry; IF immunofluorescence; DSB double-strand break; CRT calreticulin; TiME tumor immune microenvironment; DC dendritic cell. *p < 0.05; **p < 0.01; ***p < 0.001; ****p < 0.0001. The data are presented as the median and interquartile range (**c**, **d** and **g**, **h**). Comparisons were performed using one-way ANOVA with Tukey’s test for multiple comparisons. This figure was completed on the Figdraw platform (https://www.figdraw.com/)

To identify inflammatory mediators that may affect the TiME in a paracrine manner, the intersection of the differentially expressed genes obtained via transcriptome sequencing with the differentially expressed proteins obtained via proteomic analysis (Fig. 8a) was performed. Through further screening in conjunction with a literature search, we identified 2 proteins (IFN-γ and CXCL9) that are significantly associated with TIME activation (Fig. 8a), which were significantly upregulated in the UHDR-RT subgroup, as confirmed by IF and IHC (Fig. 8b–d). CXCL10 and CXCL11, which were significantly upregulated in the transcriptome, were also examined by IHC, but no significant differences were found between the groups (Supplementary Fig 9e–g). Additionally, in the endogenous antibody blockade experiments, UHDR-RT combined with anti-CSF1R significantly decreased the expression of CXCL9 compared with that in the other subgroups, whereas UHDR-RT combined with the anti-CD8A subgroup significantly decreased the expression of IFN-γ (Fig. 8b–d). IF confirmed the colocalization of M1 macrophages with CXCL9 and of CD8^+^ T cells with IFN-γ (Fig. 8e). We then further, via ELISA, verified the significant upregulation of CXCL9 and IFN-γ in vitro by assaying post-RT supernatants from BMDMs and SPTCs, respectively (Fig. 8f–h). The above results suggest that CXCL9 is secreted mainly by macrophages in the TiME, whereas IFN-γ is secreted mainly by CD8^+^ T cells.

In summary, we may conclude that UHDR-RT can directly activate CD8^+^ T cells and simultaneously induce CD8^+^ T cells to release IFN-γ, which promotes the polarization of M1-type macrophages in a paracrine manner. These polarized M1-type macrophages, in turn, further promote the activation and chemotaxis of CD8^+^ T cells by releasing CXCL9, resulting in positive feedback and achieving TiME activation (Fig. 8i).

## Discussion

To the best of our knowledge, in contrast to the extensive body of research focused on the mechanisms by which UHDR-RT protects normal tissues, our study is the first systematic demonstration and attempt to explain the reversal of HNSCC RT resistance by UHDR-RT. As we showed in this study, UHDR-RT at the same dose as Conv-RT can effectively reverse RT resistance in HNSCC by exacerbating DNA damage and activating the inhibitory TiME. Notably, we also found that this reversal of RT resistance had an abscopal effect, which has important implications for the clinical application of UHDR-RT.

Electron beams, photon beams, and proton beams can all conduct the UHDR-RT.^6,14,21–23^ However, electron irradiators have low energy (4–6 MeV) and penetration depth, limiting their clinical application to superficial tumors. Owing to the Bragg peak effect, proton rays can significantly improve the safety of RT, but the high construction and operational costs of proton UHDR-RT hinder its widespread clinical use.^24^ In 2021, the team from CAEP first reported that the advanced RT research platform (PARTER), which is based on the Chengdu Terahertz Free Electron Laser Facility, uses a superconducting linear accelerator to generate HEX (6–8 MeV) with a dose rate of ~1000 Gy/s.^6^ This is the first report of HEX-induced UHDR-RT in the literature. In contrast, through a different technical approach, our team successfully developed an ultrahigh-power rhodotron accelerator capable of conducting the HEX-based UHDR-RT, which makes it, to the best of our knowledge, the second ultrahigh-power rhodotron accelerator in the world, following the UHDR-RT platform at Aerial/Feerix produced by IBA in Belgium. As we have demonstrated, this accelerator exhibited superior performance and stability during the experiments. The rhodotron accelerator is characterized by two key features: a low instantaneous dose rate and a high average dose rate. Compared with conventional linear accelerators, a high-power rhodotron accelerator can achieve the combined average power output of multiple conventional linear accelerators. Furthermore, the rhodotron accelerator offers lower costs and reduced maintenance complexity than linear induction accelerators do. Given its advantages in cost, technological maturity, and key performance metrics, the rhodotron accelerator is well-suited for UHDR-RT experimental research.^17^

In terms of tumor control, most previous studies have shown that UHDR-RT is comparable to Conv-RT.^25–27^ Its unique advantage lies in its differential response to normal and tumor tissues (i.e., the FLASH effect). In the present study, we demonstrated for the first time the differential response of UHDR-RT to tumor tissues with different radiosensitivities to Conv-RT. Specifically, UHDR-RT not only has the same ability to control RT-sensitive tumors as Conv-RT but also has significant control efficacy for Conv-RT-resistant tumors. In this study, we systematically explored the mechanism of reversal of RT resistance by UHDR-RT using an RT-resistant cell model, a PDX model, and a C57BL/6J subcutaneous tumor model. The cellular models demonstrated a significant inhibitory effect of UHDR-RT on the malignant phenotype and DNA damage repair capacity of the MOC1_R cell line. Although the PDX model did not demonstrate better tumor control by UHDR-RT, transcriptome sequencing still suggested the inhibitory effect of UHDR-RT on DNA damage repair in RT-resistant PDX tumor tissues. These findings also suggest that the inhibition of DNA damage repair is only part of the mechanism by which UHDR-RT reverses RT resistance, and C57BL/6J mouse experiments confirmed the critical role of TiME activation in this context. In mice, UHDR-RT not only controlled the growth of RT-resistant tumor tissue within the irradiated area but also had a significant control effect on contralateral tumors. This further demonstrates that UHDR-RT primarily activates TiME to reverse RT resistance.

Previous research has shown that, compared with Conv-RT, UHDR-RT may trigger more significant immune responses, including more immune cells (such as CD8^+^ T cells) and more pronounced inflammatory responses.^26–29^ However, these reports did not study UHDR-RT based on the HEX setting, nor did they study it in the context of the RT-resistance setting. In this study, we fully clarified the critical role of macrophages and CD8^+^ T cells in activating the TiME through multiple approaches. Moreover, we clarified the relationship between the two by performing transwell and co-culture experiments. The results confirmed that UHDR-RT-induced activation of CD8^+^ T cells subsequently significantly induced the upregulation of M1 macrophages, which promoted further infiltration of CD8^+^ T cells via feedback. Moreover, UHDR-RT decreased the proportion of M2-type macrophages, which in turn led to further TiME activation. Previous studies have shown that the activation of CD8^+^ T cells is usually accompanied by the secretion of IFN-γ, which not only enhances the antitumor effect of T cells but also promotes the polarization of M1 macrophages.^30–32^ M1 macrophages have been reported to infiltrate CD8^+^ T cells by secreting CXCL9.^33,34^ Consistently, the FCM and ELISA results of this study also revealed a significant increase in IFN-γ secretion by CD8^+^ T cells and in CXCL9 secretion by M1 macrophages in the UHDR-RT group. Moreover, transcriptome sequencing revealed significant upregulation of pathways such as the JAK/STAT, NF-κB, Toll-like receptor, and NOD-like receptor pathways, suggesting that CD8^+^ T cells may promote the activation of M1 macrophages through these pathways.

As reported by Eggold et al., UHDR-RT could increase intratumoral CD8^+^ T-cell infiltration in combination with PD-1 blockade therapy in an ovarian cancer model in both anti-PD-1 antibody-resistant and anti-PD-1 antibody-sensitive settings.^29^ Similarly, PD-1 monoclonal antibodies combined with UHDR-RT significantly reduced the tumor volume in the MOC1_R mouse model. These findings demonstrate the synergistic therapeutic potential of UHDR-RT and other immune-enhancing strategies and may provide a pathway to reverse drug resistance in patients receiving anti-PD-1 immunotherapy, with very important clinical implications.

One of the limitations of this study that must be acknowledged is the significant correlation between HPV infection status and treatment resistance in HNSCC, as reported in previous studies.^35,36^ This correlation may be attributed to differences in cancer stem cell phenotypes and tumor microenvironment characteristics.^35,36^ However, due to the space constraints of this manuscript and the specific nature of the models used, we were unable to explore this issue in detail. In addition, it must be noted that the radioresistant cell and animal models used in this study were all secondary radiotherapy resistance models. Whether UHDR-RT can reverse resistance in the context of primary radiotherapy resistance remains an interesting question. We will further explore this issue in future studies.

In summary, our study is the first to discover and validate the significant phenomenon that UHDR-RT can reverse RT resistance in HNSCC. We also, for the first time, comprehensively elucidate and validate the underlying mechanisms by which UHDR-RT reverses RT resistance through increasing DNA damage and activating the TiME. Our findings provide biological support for accelerating the clinical application of UHDR-RT via the rhodotron accelerator. Moreover, this study highlights the potential of rhodotron accelerator-based UHDR-RT and offers new therapeutic strategies and approaches for addressing RT resistance in HNSCC.

## Materials and methods

### Experimental design

This study aimed to investigate the differences in antitumor immune responses between ultra-high dose rate radiotherapy (UHDR-RT) and Conv-RT, with a specific focus on the impact of radiation on the microenvironment of radioresistant tumors. The design includes two main experimental groups, UHDR-RT and Conv-RT, ensuring that the irradiation conditions (dose distribution, electron energy, and peak power) are identical to eliminate the influence of these factors on the results. Predefined components of the experiment include the construction of cell lines, the establishment of animal models, the preparation of tumor-conditioned media, and associated functional and molecular analyses. All the experiments adhered to random assignment principles and underwent thorough statistical analysis to ensure the reliability and reproducibility of the findings.

### The rhodotron accelerator and dosimetry

The rhodotron accelerator was independently developed by the Institute of Fluid Physics of CAEP, whose electron energy at the exit is 8 MeV with an adjustable average current from 0.1 µA to 3 mA and an energy spread of less than 1%, and whose peak beam power is ~40 kW.^17^ Both UHDR-RT and Conv-RT irradiation were performed on the same platform; therefore, the dose distribution, electron energy, and peak power of the electron beam were the same, eliminating the contributions of these factors to the comparison results.

The absolute standard dose was set via a medical linear accelerator (Synergy, Elekta, Sweden) in the Radiotherapy Department of West China Hospital, and the dose was determined via the same batch of Gaf-chromium films (EBT4, Ashland, USA). Twenty-four hours after exposure, the films were scanned (11000XL, Epson, Japan), and a dose calibration curve was created via SNC Patient software (Sun Nuclear, USA). In this study, uniformity within the irradiated area was assessed using the RMS deviation (*U*_rms_), defined as ${U}_{{\rm{rms}}}={\rm{RMS}}(D)$, where *D* represents the dose. For Gaussian error distributions, ${U}_{{\rm{rms}}}\approx 1/6\times ({D}_{\max }-{D}_{\min })$, where *D*_max_ and *D*_min_ represent the maximum and minimum doses within the irradiated area.

### Cell experiments

Human CAL33 (ATCC) and mouse oral squamous cell carcinoma MOC1 (ATCC) cell lines were cultured in Roswell Park Memorial Institute 1640 medium (#C11875-500BT, Gibco, USA) and Dulbecco’s modified Eagle’s medium (DMEM) (#C11995-500BT, Gibco, USA) with 10% fetal bovine serum (FBS) (#Z7185FBS-500, ZETA, USA). RT-resistant cell lines were established by irradiating cells with 2 Gy X-rays (RS2000, Rad Source Technologies, USA) five times per week until a cumulative dose of 70 Gy was reached. Bone marrow-derived macrophages (BMDMs) were differentiated from 6 to 8 weeks old male C57BL/6J mice bone marrow cells using 20 ng/mL macrophage colony-stimulating factor (M-CSF) (#315-02, Peprotech, USA) for 7 days. Tumor-associated macrophages (TAMs) were differentiated by adding tumor tumor-conditioned medium (CM) for 24 h. Naïve T cells were extracted from spleens of 6–8 weeks old male C57BL/6J mice, stimulated with CD3 (#16-0281-82, eBioscience, USA), CD28 (#16-0031-81, eBioscience, USA), and IL-2 (#212-12, Peprotech, USA) to obtain mature T cells. Cells were maintained at 37 °C in a 5% CO_2_ incubator.

Cell proliferation was measured using the Cell Counting Kit-8 (CCK-8) assay (#CK04, Dojindo, Japan), and analyzed with a multi-functional microplate reader (SYNERGY H1, Bio-Tek, USA). Cell migration was assessed using the scratch test (AutoScratch instrument, BioTek, USA). Cell invasion was evaluated with the transwell assay (chamber: Corning #3422; matrix gel: Beyotime #C0372). Cell viability was determined using the Calcein/PI cell viability/cytotoxicity assay kit (#C2015S, Beyotime Biotechnology, Shanghai, China). DNA damage was assessed with the γ-H2AX immunofluorescence assay kit (#C2035, Beyotime Biotechnology). Mitochondrial membrane potential was measured using the JC-1 assay kit (#C2006, Beyotime Biotechnology). Observations were made with a fluorescence microscope (VS200, Olympus, Japan).

For cell colony assay, cells were seeded into six-well plates at a density of 1000 cells per well and cultured under standard conditions (37 °C, 5% CO_2_) for 14 days. The cells were then fixed with 4% paraformaldehyde and stained with crystal violet to visualize. Images of the fixed and stained plates were captured using a digital camera and analyzed with Image J software to measure the colonies number. The “Set Scale” function was used to calibrate the actual dimensions of the image. The “Threshold” tool was then applied to differentiate the colony regions from the background, ensuring accurate identification of all colonies. Using the “Analyze Particles” function, the number of colonies was recorded, and appropriate thresholds were set to exclude small particles or background noise. The resulting data were extracted from the output table, and the total colonies number was calculated to evaluate the colony-forming ability of the cells. Fit the cell survival curve using the following equation: survival fraction (SF) = exp (−*αD* − *βD*²).

### Animal experiments

All Animal experiments in this study have already been approved by the ethical committee of West China Hospital of Sichuan University. Specifically, the ethical approval number for the PDX model construction experiment is No. 20211673.

For the PDX model, 6-week-old BALB/C-nude mice (Beijing HFK Bioscience) were implanted with human laryngeal cancer tissues. Tumor tissues were trimmed, transplanted subcutaneously, and stabilized for three passages before use when tumors reached 100 ± 25 mm³.

The SPF female C57BL/6J mice (Beijing HFK Biotechnology) were used for HNSCC model development. MOC1 or MOC1_R cells (1 × 10^6^) were injected subcutaneously. Treatments with anti-PD-1 monoclonal antibody (10 mg/kg) (RMP1–14, Selleck, USA), anti-CD8a monoclonal antibody (10 mg/kg) (BE0004-1, BioXcell, USA), and anti-CSF1R monoclonal antibody (25 mg/kg) (BE0213, BioXcell, USA) were administered intraperitoneally with specified dosing schedules. Tumor volume was calculated using calipers, and animals were euthanized for tumor analysis.

### RNA-sequencing, Olink proximity extension assay (PEA), and weighted gene co-expression network analysis (WGCNA)

Total RNA was extracted using TRIzol (#15596018, Thermo Fisher, USA), and RNA quality was assessed with a NanoDrop ND-1000 spectrophotometer (NanoDrop, Wilmington, USA) and Bioanalyzer 2100 (Agilent, USA). Suitable RNA samples were sequenced on an Illumina NovaSeq™ 6000 (LC Bio-Technology CO., Ltd., China) using paired-end 150 bp mode (PE150) as previously reported.^37^ Protein levels were measured with the Olink Target 48 Mouse panel (Olink Proteomics AB, Sweden). The PEA technology used for the Olink protocol has been well described.^38,39^ The Target 48 Mouse panel included 48 proteins in important immune and inflammation pathways, as listed in Supplementary Table 1. The WGCNA analysis was performed using OmicStudio tools (https://www.omicstudio.cn/tool/WGCNA) with parameters set to a soft threshold power of 0.85.

### Flow cytometry (FCM)

The CytoFLEX flow cytometer (Beckman Coulter, USA) was used with the Annexin V-FITC Apoptosis Detection Kit (#C1062M, Beyotime Biotechnology, China) and the Reactive Oxygen Species Assay Kit (#S0033, Beyotime Biotechnology, China). Mouse immune cells, BMDMs, and SPTCs samples were analyzed using spectral flow cytometry (Aurora CS, Cytek, USA) with a panel of antibodies including Ms CD16/CD32 Pure 2.4G2, APC-Cy7 Rat Anti-Mouse CD5 (30-F11), Fixable Viability Stain 440UV, BV395 Hamster Anti-Mouse CD3e (145-2C11), BUV661 Rat Anti-Mouse CD4 (RM4-5), BV711 Rat Anti-Mouse CD8a (53-6.7), BV750 Mouse Anti-Mouse NK-1.1 (PK136), BV421 Rat Anti-CD11b (M1/70), BV605 Rat Anti-Mouse F4/80 (T45-2342), BUV737 Rat Anti-Mouse CD86 (PO3), Alexa Fluor 647 Rat Anti-Mouse CD206 (MR5D3), BUV563 Hamster Anti-Mouse CD11c (HL3), and R718 Rat Anti-Mouse I-A/I-E (2G9), all from BD Pharmingen.

### Immunohistochemical staining (IHC) and immunofluorescence (IF)

Cells were stained using a rabbit anti-calreticulin/AF488 conjugated antibody (Bioss, China) to detect the expression of calreticulin. Tumor specimens were fixed in 10% neutral-buffered formaldehyde and embedded in paraffin. Sections (4 µm thick) were deparaffinized, rehydrated, and subjected to antigen retrieval using Tris-EDTA buffer (pH 8.0). Sections were blocked with 10% normal goat serum. Primary antibodies included anti-mouse CXCL9 (#22355-1-AP, Proteintech, USA), CXCL10 (#10937-1-AP, Proteintech, USA), CXCL11 (#10707-1-AP, Proteintech, USA), CD8 (#GB15068, Servicebio, China), CD86 (#GB115630, Servicebio, China), and CD206 (#GB113497, Servicebio, China). Sections were incubated with secondary antibodies conjugated to horseradish peroxidase or fluorophores, and stained with DAPI for nuclei visualization. Fluorescence microscopy was performed with VS200 (Olympus, Japan) and IHC was detected using DAB chromogen, observed with Axio Imager A2 LED/DL (Zeiss, Germany).

### Construction of co-culture system

BMDMs and SPTCs (1 × 10^6^ cells per well) were cultured in different CM (Ctrl, MOC1, MOC1_R) and irradiation conditions (Ctrl, Conv-RT, UHDR-RT). Co-culture was performed in 24-well plates and transwell chambers (0.4 µm and 5 µm pores; #3470, Corning, USA; #14331, Labselect, China). The migratory capacity of macrophages was assessed by counting the cells that remained on the membrane and were stained with crystal violet. The migratory capacity of T cells was evaluated based on the number of cells that penetrated the membrane and were present in the lower culture medium.

### Cell supernatant extraction and enzyme-linked immunosorbent assay (ELISA)

Supernatants from CAL33 and CAL33_R were analyzed for calreticulin and HMGB1 secretion levels using ELISA kits: human calreticulin ELISA Kit (#BP01643, Baipeng, China) and human HMGB1 ELISA Kit (#HM10235, Bioswarmp, China). Supernatants from BMDMs and SPTCs were analyzed for CXCL9 and IFN-γ secretion levels using ELISA kits: mouse CXCL9 ELISA Kit (#MU30720, Bioswarmp, China) and mouse IFN-γ ELISA Kit (#MU30038, Bioswarmp, China).

### ATP detection and chemiluminescence assay

The cell supernatants were collected to assess extracellular ATP release. Subsequently, the cells were lysed for the determination of intracellular residual ATP. All procedures were performed in accordance with the protocol provided by the ATP Detection Kit (#S0027, Beyotime Biotechnology). Chemiluminescence intensity was quantitatively measured using a multifunctional microplate reader (PE EnVision, PerkinElmer, USA).

### Statistical analysis

Statistical analyses were conducted using GraphPad Prism 9.5 (San Diego, USA). Data are presented as the mean ± SEM of at least three independent experiments. Two-tailed unpaired Student’s *t* test and one-way or two-way ANOVA with Tukey’s test for multiple comparisons were used to calculate *P* values. Significance was set at *P* < 0.05. Mechanism graphs were created using the Figdraw platform (https://www.figdraw.com/#/).

## Supplementary information

Supplementary Materials

## Data Availability

The transcriptome sequencing data generated in this study have been deposited in the NCBI Sequence Read Archive (SRA) under BioProject accession number PRJNA1201890. The Olink proteomics data was provided in Supplementary materials. Additional datasets supporting the findings of this study are available from the corresponding authors upon reasonable request.
