# Supplementary material for: Ultra-high dose rate radiotherapy overcomes radioresistance in head and neck squamous cell carcinoma
Source: Signal Transduct Target Ther. 2025 Mar 3;10:82. doi: 10.1038/s41392-025-02184-0 (PMC11876629; doi:10.1038/s41392-025-02184-0)
Supplement: Supplementary file 1 — Supplementary Materials [file 41392_2025_2184_MOESM1_ESM.docx]

Supplementary Materials for

• Ultra-High Dose Rate Radiotherapy Overcomes Radioresistance in Head and Neck Squamous Cell Carcinoma by Exacerbating DNA Damage and Activating the Tumor Immune Microenvironment

Hong-Shuai Li†; Ruo Tang†; Hua-Shan Shi†; Zi-Jian Qin; Xiao-Yang Zhang; Yun-Fei Sun; Zhi-Gong Wei; Chao-Fan Ma; Liu Yang; Ye Chen; Zhe-Ran Liu; Li-Li Zhu; Wen Yang; Li-Yang; Ai-Ning Xu; Zhuo Zhang; Shu-Qing Liao; Jin-Shui Shi; Jian-Jun Deng; Xiao-Zhong He*; Xing-Chen Peng*

†These authors contributed equally to this work.

Correspondence to: Xing-Chen Peng (pxx2014@163.com) and Xiao-Zhong He (hexiaozhong@caep.cn).

**This PDF file includes:**

Figures. S1 to S9

Tables S1 to S3

**Other Supplementary Materials for this manuscript include the following:**

None.


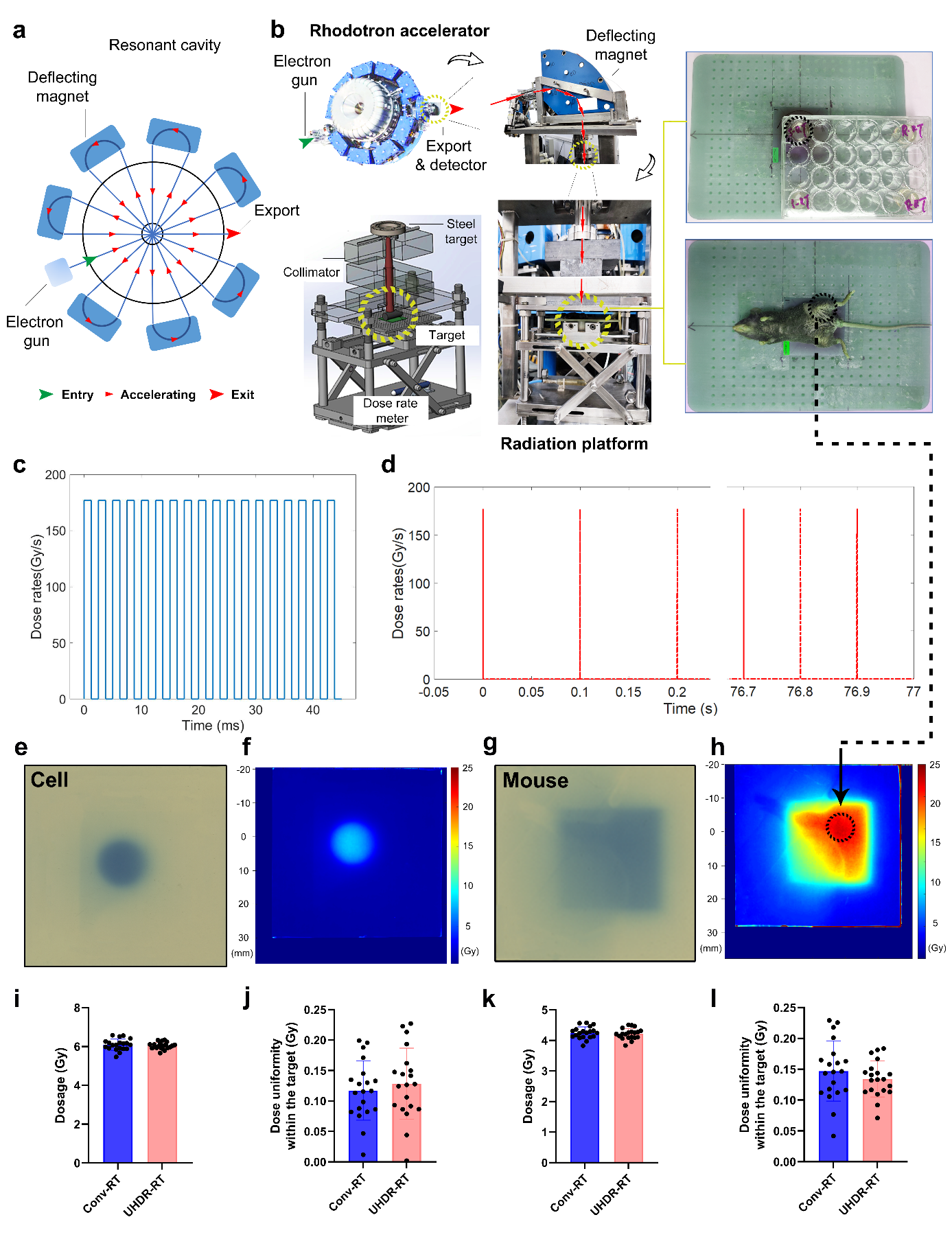


Figure. S1.

**The** **rhodotron accelerator shows good performance and beam output quality.** The structure schematic of the rhodotron accelerator (**a**) and radiation platform (**b**). The fixation method for cell samples and animal samples were also displayed (**b**). Display of radiation time structure and dose rate for UHDR-RT (**c**) and Conv-RT (**d**). Dose distribution in the radiation area of cell samples (**e-f**). Dose distribution in the radiation area of mouse samples (**g-h**). Dose administration and dose distribution during cell (**i-j**) and animal experiments (**k-l**). Ctrl, control; Conv-RT, conventional radiotherapy; UHDR-RT, ultra-high dose rate radiotherapy; CCK-8, cell counting kit-8; ns, not statistically significant. The red arrows represent the direction of the beam transmission. The yellow dotted circles represent the irradiation platform and the target. The black dotted circles represent the tumor locations in the mice and their corresponding positions on the film. *, p＜0.05; **, p＜0.01; ***, p＜0.001; ****, p＜0.0001. The data are presented as the mean ± SD (**i-l**). Comparisons were performed using two-tailed unpaired Student’s t test.


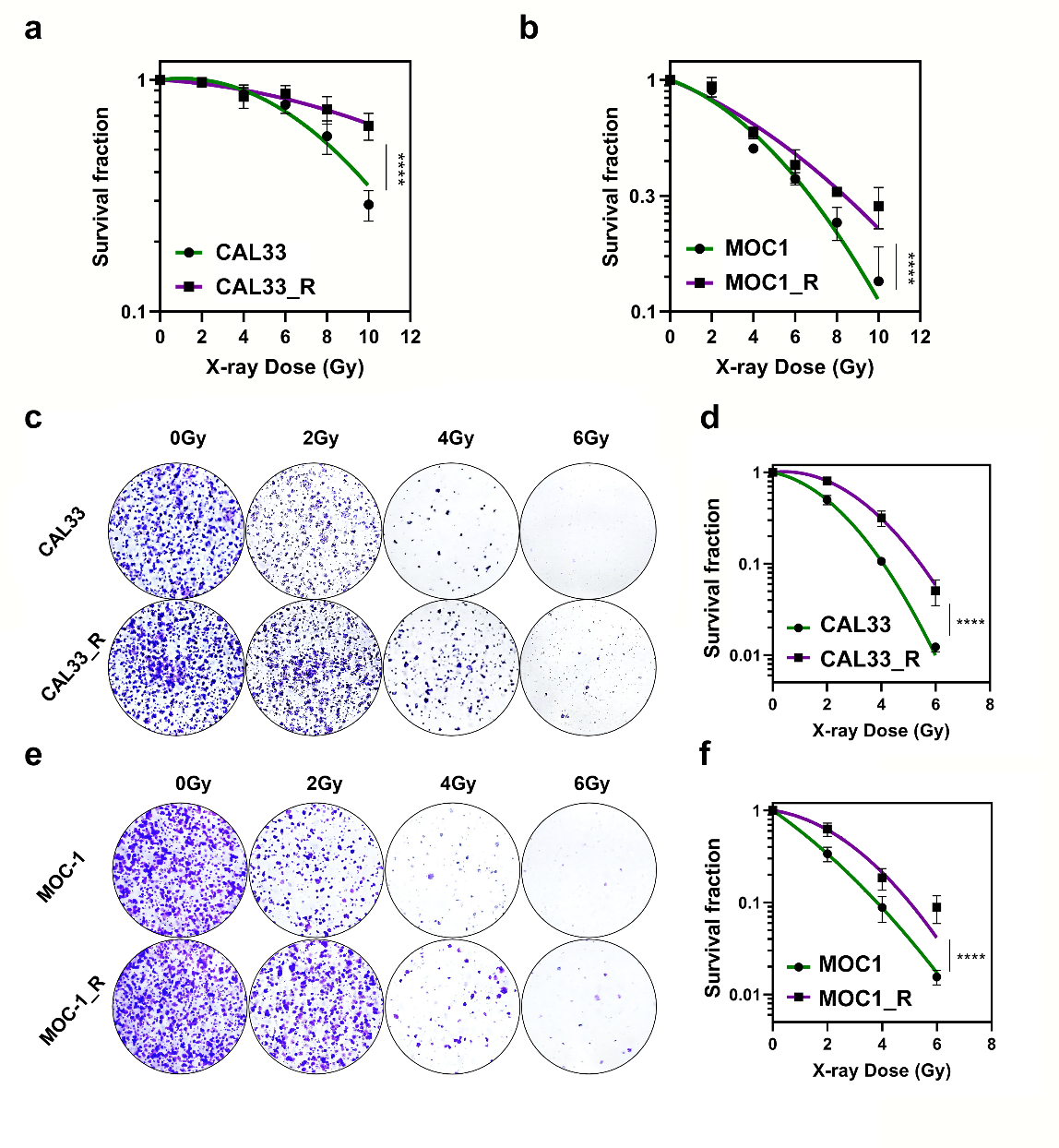


Figure. S2.

**Identification of RT-resistant cell lines.** The 72-hour CCK-8 assays (**a-b**) confirmed the RT-resistance of CAL33_R (**a**) and MOC1_R (**b**), while the colony formation assays demonstrated the enhanced colony-forming ability of CAL33_R (**c-d**) and MOC1_R (**e-f**). RT, radiotherapy; CCK-8, cell counting kit-8. *, p＜0.05; **, p＜0.01; ***, p＜0.001; ****, p＜0.0001. The data are presented as the mean ± SEM (**a-b**, **d**, and **f**). Comparisons were performed using two-way ANOVA method.


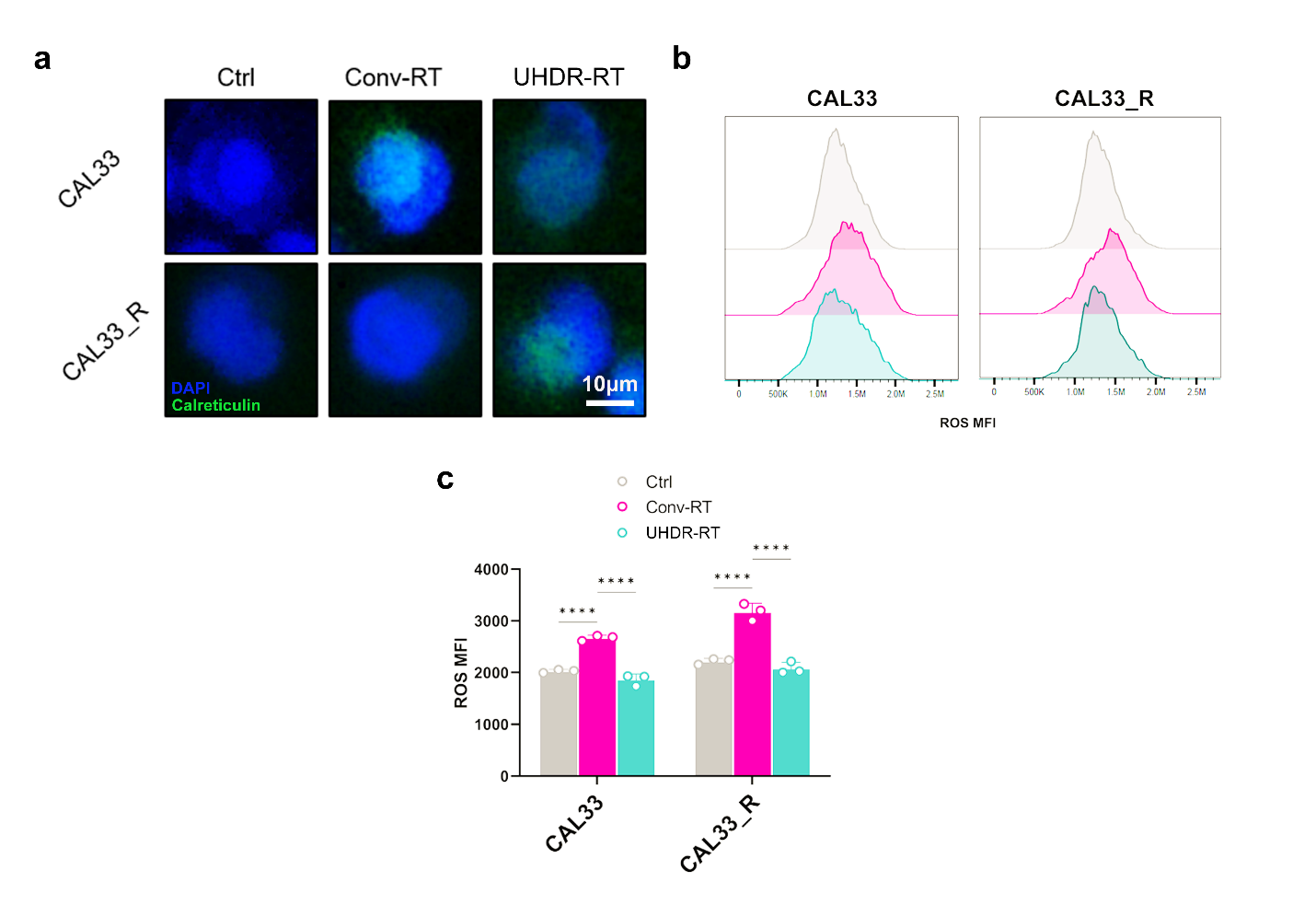


Figure. S3.

**UHDR-RT increases the levels of the ICD marker calreticulin and reduces ROS levels compared to Conv-RT.** Immunofluorescence staining indicated the expression of calreticulin (**a**). The median MFI of ROS was detected by FCM (**b-c**). ROS, reactive oxygen species; MFI, mean fluorescence intensity; Ctrl, control; Conv-RT, conventional radiotherapy; UHDR-RT, ultra-high dose rate radiotherapy; ICD, immunogenic cell death; FCM, flow cytometry. *, p＜0.05; **, p＜0.01; ***, p＜0.001; ****, p＜0.0001. All data are presented as the mean ± SEM. Comparisons were performed using one-way ANOVA with the Tukey’s test for multiple comparisons.


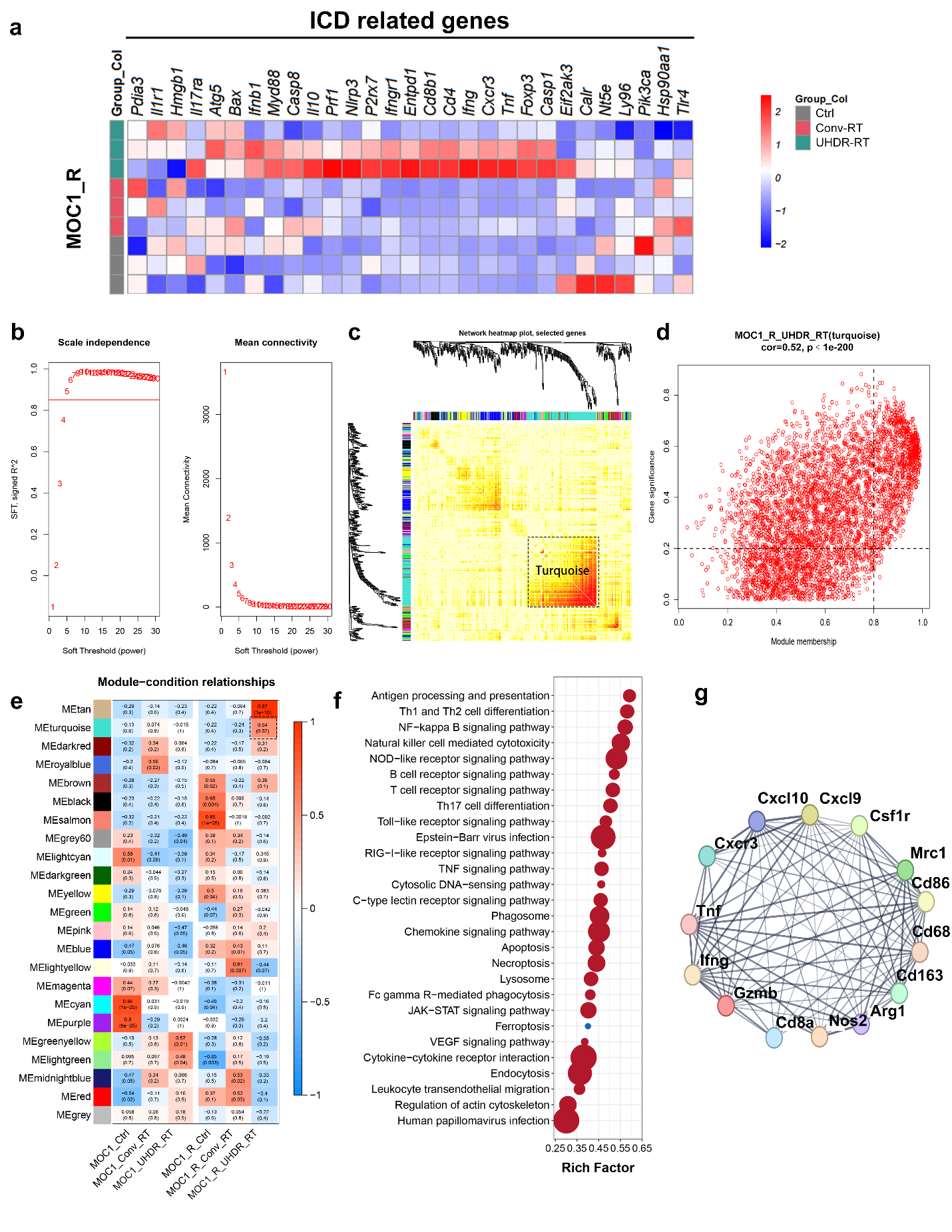


Figure. S4.

**The ICD related genes expression profile and the WGCNA analysis.** MOC1_R tumors receiving UHDR-RT showed higher levels of ICD related genes expression **(a)**. The plateau threshold line set for this WGCNA analysis was 0.85, and the soft threshold was 5 (**b**). The gene co-expression network heatmap showed the effect of WGCNA module delineation, and the heatmap region indicated the dissimilarity between genes, with smaller values being darker in color (**c**). We then calculated the correlation between modules and samples (**d**), and plotted the correlation heatmap to identify the modules with the highest degree of correlation with samples (**e**). KEGG enrichment analysis showed that the UHDR-RT subset of the MOC1_R group was significantly enriched and immune-related pathways (**f**). The correlation network diagram showed the interaction of key genes within the modules (**g**). Ctrl, control; Conv-RT, conventional radiotherapy; UHDR-RT, ultra-high dose rate radiotherapy; ICD, immunogenic cell death. The correlation analysis was performed using Pearson’s method.


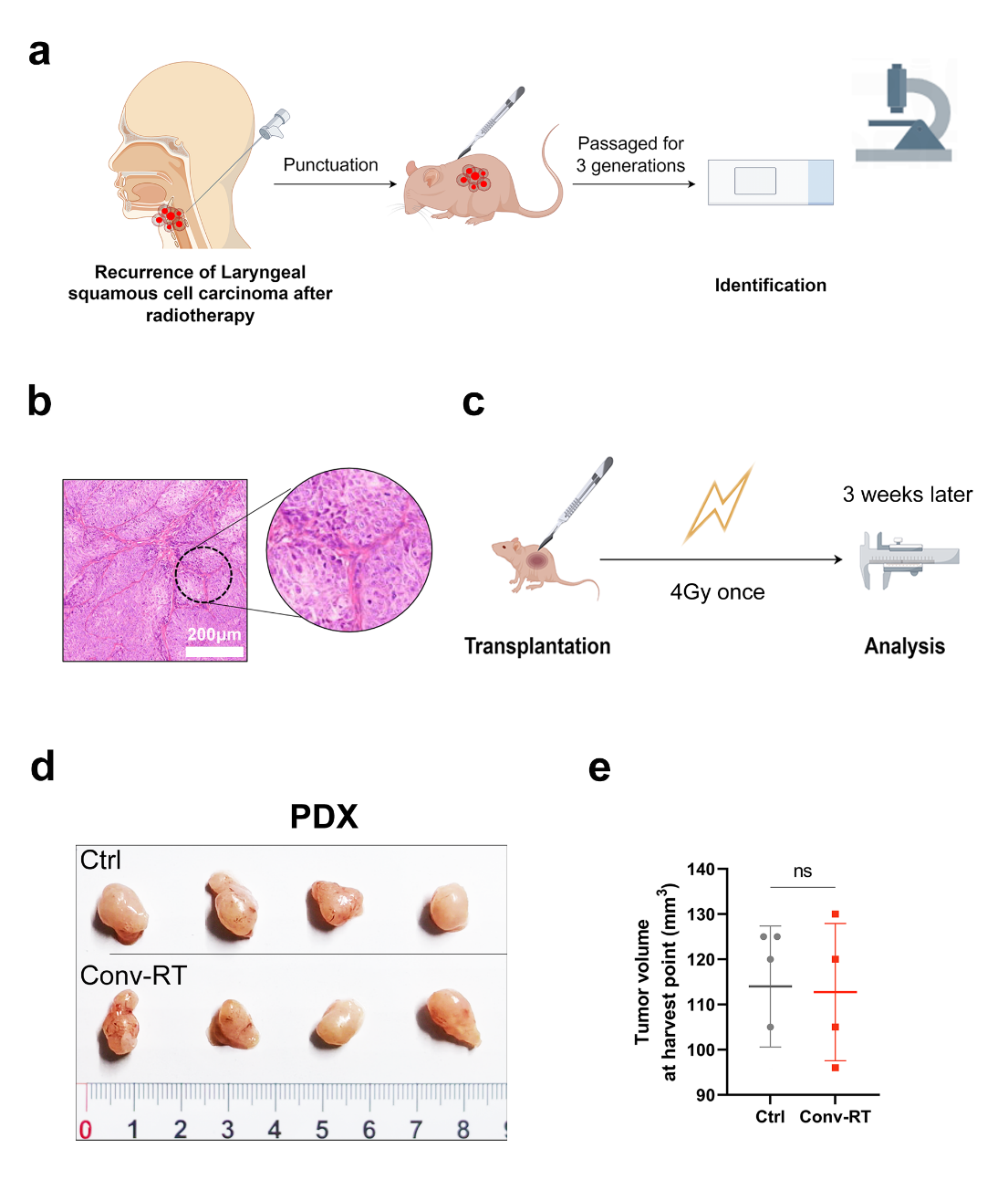


Figure. S5.

**Construction and characterization of the PDX model.** The process of constructing the PDX model is shown (**a**). HE staining indicates the typical histological structure of squamous cell carcinoma (**b**). The animal experiments showed that the tumor volume in the PDX model (n=4) receiving Conv-RT did not differ significantly from the Ctrl group (PDX model without Conv-RT, n=4) (**c-e**). Ctrl, control; Conv-RT, conventional radiotherapy; PDX, patient-derived xenograft; HE staining, hematoxylin-eosin staining. ns, not statistically significant. The data are presented as the mean ± SD. Comparisons were performed using two-tailed unpaired Student’s t test.


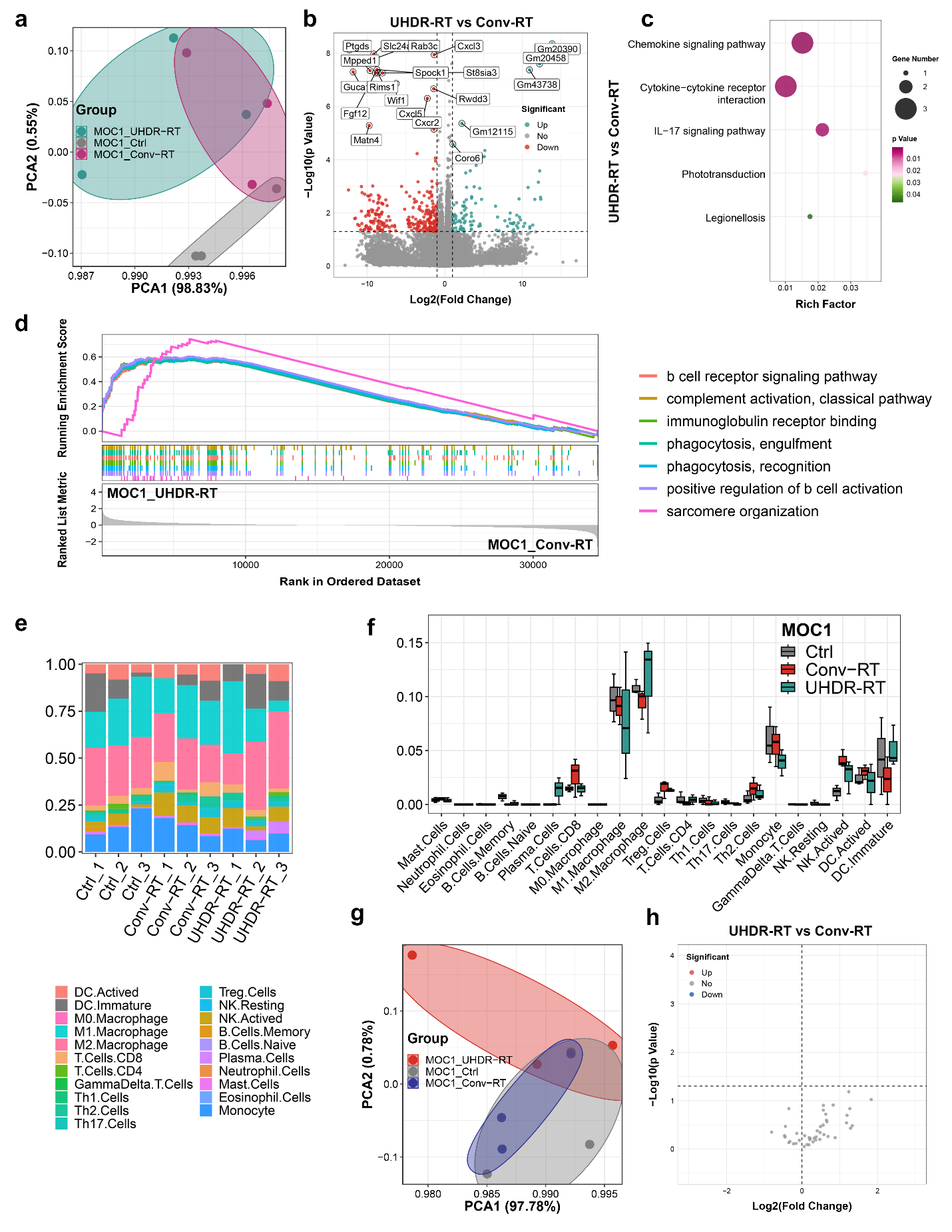


Figure. S6.

**Combined transcriptomic and proteomic analysis of RT-sensitive group.** Transcriptome PCA analysis shows the MOC1 group subgroup (**a**). Volcano plots showing significantly expressed differential genes for UHDR-RT vs. Conv-RT in the MOC1 subgroups (**b**). KEGG analysis and GSEA demonstrating enrichment pathways in the UHDR-RT subgroup (**c-d**). Cybersort analysis demonstrating immune cell distributions of subgroups within the MOC1 group (**e-f**). Proteomic PCA analysis of subgroups (**g**). Volcano plot demonstrating differential genes in UHDR-RT versus Conv-RT subgroups (**h**). Ctrl, control; Conv-RT, conventional radiotherapy; UHDR-RT, ultra-high dose rate radiotherapy. The data are presented as the median and interquartile range (**f**). The boxplots indicate median (center), 25th and 75th percentiles (bounds of box), and minimum and maximum (whiskers). Comparisons were performed using one-way ANOVA method.


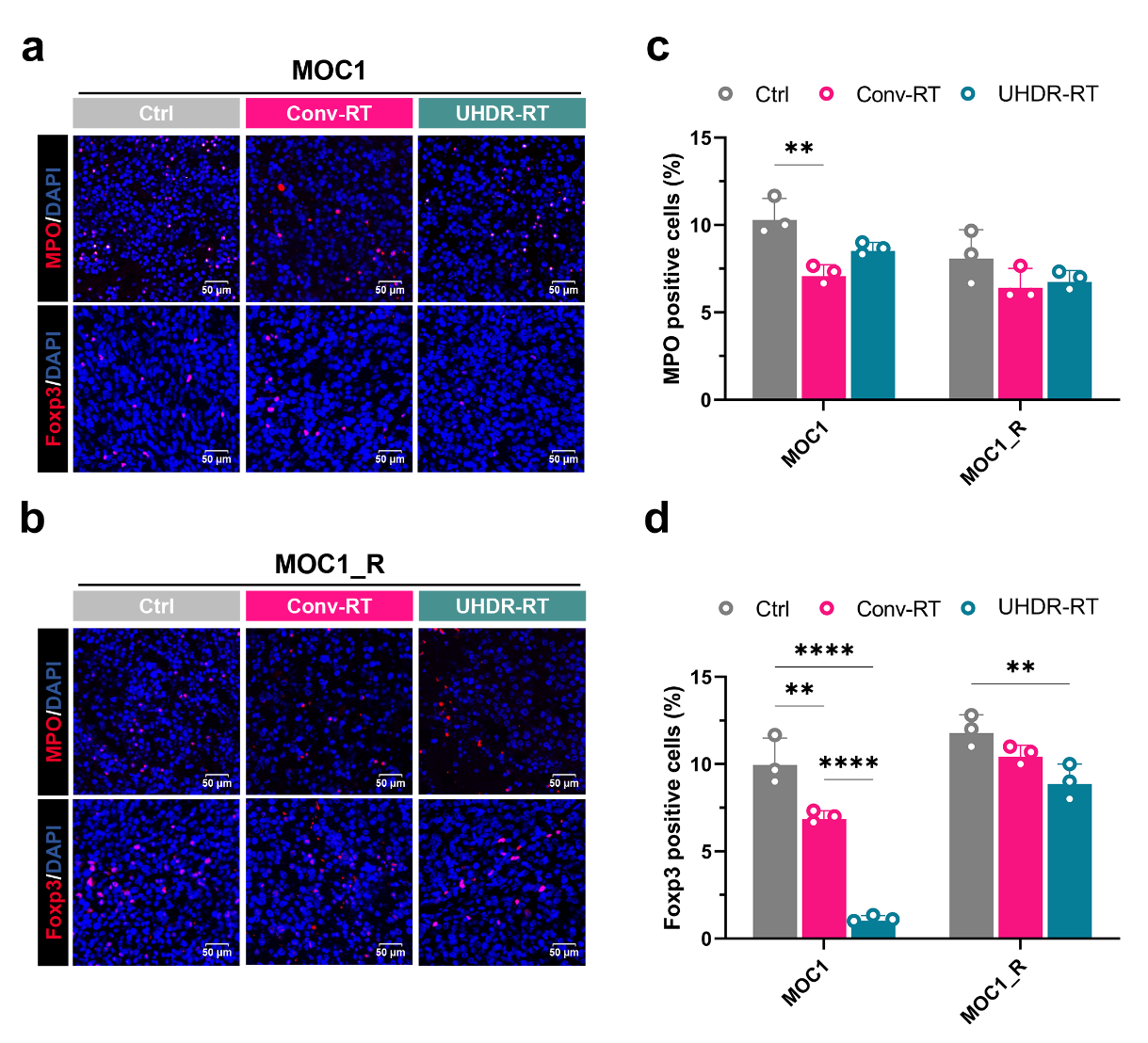


Figure. S7.

**Single-color IF of Foxp3 and MPO.** Single-color IF showed, in MOC1 (**a**) and MOC1_R (**b**), no significant differences in neutrophil marker (MPO) (**c**) and Treg marker (Foxp3) (**d**) between the UHDR-RT and Conv-RT subgroups. Ctrl, control; Conv-RT, conventional radiotherapy; UHDR-RT, ultra-high dose rate radiotherapy; MPO, Myeloperoxidase; IF, immunofluorescence. *, p＜0.05; **, p＜0.01; ***, p＜0.001; ****, p＜0.0001. The data are presented as the mean ± SEM (**c-d**). Comparisons were performed using one-way ANOVA with the Tukey’s test for multiple comparisons.


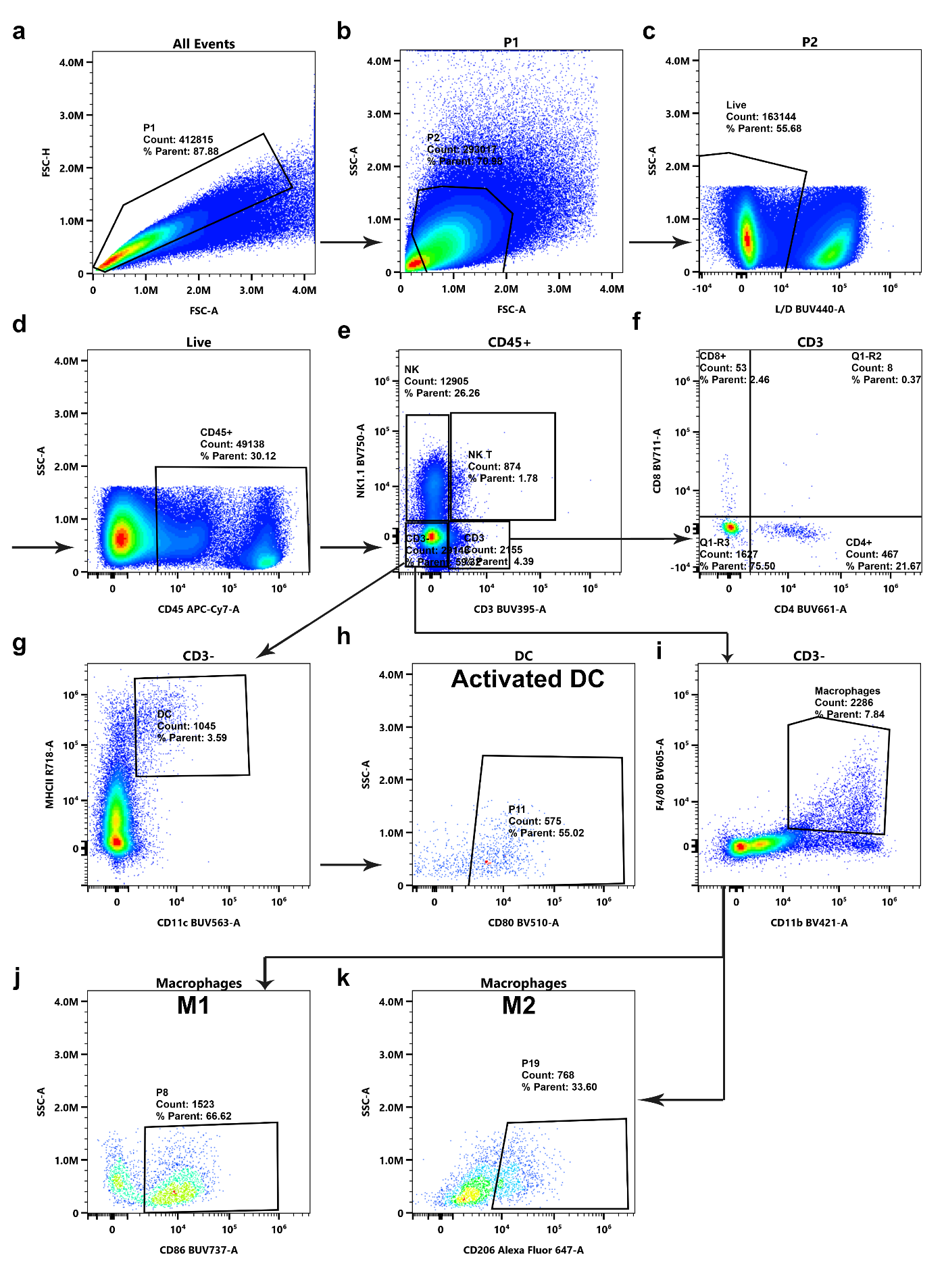


Figure. S8.

**Tumor tissue and spleen multi-color FCM gating strategy.** DC, dendritic cells


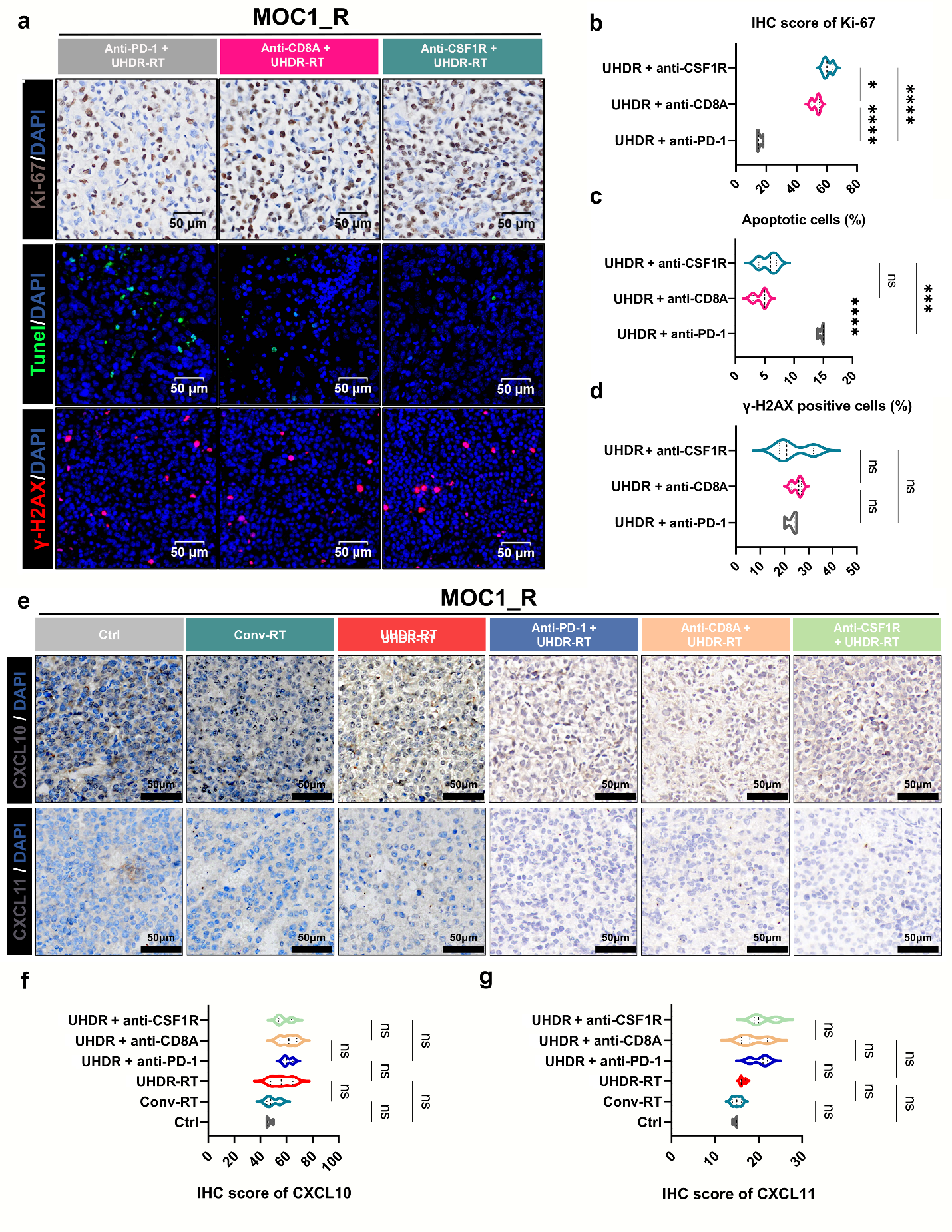


Figure. S9.

**IHC and IF results from mouse tumor tissues.** Ki-67 staining, Tunel staining and DNA damage staining of different treatment modalities in MOC1_R group (a-d). CXCL10 and CXCL11 IHC staining of different treatment modalities in MOC1_R group (e-g). Ctrl, control; Conv-RT, conventional radiotherapy; UHDR-RT, ultra-high dose rate radiotherapy; IHC, immunohistochemistry. *, p＜0.05; **, p＜0.01; ***, p＜0.001; ****, p＜0.0001. The data are presented as the median and interquartile range (**b-d** and **f-g**). Comparisons were performed using one-way ANOVA with the Tukey’s test for multiple comparisons.

Table S1. The dosage accuracy and uniformity

| **Category** | **Absolute dose (Gy)** | **Dose uniformity within the target (Gy)** |
| --- | --- | --- |
| **Tumor cell experiments** (6 Gy) |  |  |
| UHDR-RT | 6.05 ± 0.18 | 0.13 ± 0.03 |
| Conv-RT | 6.09 ± 0.29 | 0.15 ± 0.05 |
| **Macrophage experiments** (4Gy) |  |  |
| UHDR-RT | 4.00±0.28 | 0.12±0.03 |
| Conv-RT | 4.18±0.18 | 0.10±0.04 |
| **T cell experiments** (2Gy) |  |  |
| UHDR-RT | 2.05±0.12 | 0.08±0.01 |
| Conv-RT | 2.07±0.14 | 0.09±0.02 |
| **Mouse experiments** (4 Gy) |  |  |
| UHDR-RT | 4.22±0.18 | 0.13±0.06 |
| Conv-RT | 4.25±0.19 | 0.12±0.05 |

UHDR-RT, ultra-high dose rate radiotherapy; Conv-RT, conventional radiotherapy. The data are presented as mean±SD.

Table S2. The Olink Target 48 Mouse panel

| **UniProt** | **Description** |
| --- | --- |
| P10148 | C-C motif chemokine 2 (Ccl2) |
| P30882 | C-C motif chemokine 5 (Ccl5) |
| P01587 | Granulocyte-macrophage colony-stimulating factor (Csf2) |
| P12850 | Growth-regulated alpha protein (Cxcl1) |
| P18340 | C-X-C motif chemokine 9 (Cxcl9) |
| Q08048 | Hepatocyte growth factor (Hgf) |
| P18893 | Interleukin-10 (Il10) |
| Q62386 | Interleukin-17A (Il17a) |
| Q7TNI7 | Interleukin-17F (Il17f) |
| P01582 | Interleukin-1 alpha (Il1a) |
| P10749 | Interleukin-1 beta (Il1b) |
| P04401 | Interleukin-5 (Il5) |
| P08505 | Interleukin-6 (Il6) |
| P06804 | Tumor necrosis factor (Tnf) |
| P48298 | Eotaxin (Ccl11) |
| Q62401 | C-C motif chemokine 12 (Ccl12) |
| Q9WUZ6 | C-C motif chemokine 17 (Ccl17) |
| O88430 | C-C motif chemokine 22 (Ccl22) |
| P14097 | C-C motif chemokine 4 (Ccl4) |
| Q9EP73 | Programmed cell death 1 ligand 1 (Cd274) |
| P07141 | Macrophage colony-stimulating factor 1 (Csf1) |
| P09920 | Granulocyte colony-stimulating factor (Csf3) |
| P09793 | Cytotoxic T-lymphocyte protein 4 (Ctla4) |
| Q9JHH5 | C-X-C motif chemokine 11 (Cxcl11) |
| P40224 | Stromal cell-derived factor 1 (Cxcl12) |
| P10889 | C-X-C motif chemokine 2 (Cxcl2) |
| Q9JJN1 | Fibroblast growth factor 21 (Fgf21) |
| P01573 | Interferon alpha-2 (Ifna2) |
| P01580 | Interferon gamma (Ifng) |
| Q4VK74 | Interferon lambda-2 (Ifnl2) |
| P43431_P43432 | Interleukin-12 (Il12a_Il12b) |
| O54824 | Pro-interleukin-16 (Il16) |
| P04351 | Interleukin-2 (Il2) |
| Q9ES17 | Interleukin-21 (Il21) |
| Q9JJY9 | Interleukin-22 (Il22) |
| Q8K3I6 | Interleukin-27 subunit alpha (Il27) |
| P01586 | Interleukin-3 (Il3) |
| Q6EAL8 | Interleukin-31 (Il31) |
| Q8BVZ5 | Interleukin-33 (Il33) |
| P07750 | Interleukin-4 (Il4) |
| P10168 | Interleukin-7 (Il7) |
| P15247 | Interleukin-9 (Il9) |
| Q9WUL5 | Programmed cell death 1 ligand 2 (Pdcd1lg2) |

Olink, Olink Proteomics.

Table S3. The Olink normalization protein expression

| **Assay** | **Uniprot ID** | **MOC1_Ctrl_1** | **MOC1_Ctrl_2** | **MOC1_Ctrl_3** | **MOC1_Conv-RT_1** | **MOC1_Conv-RT_2** | **MOC1_Conv-RT_3** | **MOC1_UHDR_RT_1** | **MOC1_UHDR_RT_2** | **MOC1_UHDR_RT_3** | **MOC1_R_Ctrl_1** | **MOC1_R_Ctrl_2** | **MOC1_R_Ctrl_3** | **MOC1_R_Conv-RT_1** | **MOC1_R_Conv-RT_2** | **MOC1_R_Conv-RT_3** | **MOC1_R_UHDR_RT_1** | **MOC1_R_UHDR_RT_2** | **MOC1_R_UHDR_RT_3** |
| --- | --- | --- | --- | --- | --- | --- | --- | --- | --- | --- | --- | --- | --- | --- | --- | --- | --- | --- | --- |
| Cxcl1 | P12850 | 12.02171 | 12.91339 | 11.3037 | 14.16519 | 12.70429 | 12.82519 | 13.73371 | 12.3352 | 12.22657 | 14.40661 | 8.48215 | 14.33045 | 14.21315 | 13.49661 | 13.92794 | 13.75026 | 13.88306 | 12.40971 |
| Il4 | P07750 | 0.33012 | 0.7257 | 1.37782 | 1.20066 | 0.73995 | 1.54882 | 1.27779 | 0.83274 | 3.1131 | 0.3456 | 1.26751 | 0.73866 | 1.36281 | 1.45777 | 0.59585 | 1.67955 | 1.33652 | 0.68778 |
| Il6 | P08505 | 5.47683 | 4.36439 | 3.67822 | 5.5458 | 4.10608 | 5.36271 | 5.17941 | 4.8309 | 5.3969 | 6.59114 | 5.72168 | 4.84907 | 5.67701 | 4.68533 | 5.62234 | 4.27333 | 5.26824 | 4.94364 |
| Fgf21 | Q9JJN1 | 10.84884 | 11.45022 | 10.82096 | 10.30768 | 7.97693 | 10.7403 | 9.83029 | 8.54417 | 9.59858 | 10.97645 | 6.76259 | 11.53292 | 9.983 | 10.76112 | 9.93395 | 9.24604 | 11.26918 | 10.65784 |
| Il21 | Q9ES17 | 0.18592 | 0.54172 | 0.25043 | -0.05739 | 0.95888 | -0.03794 | 0.5362 | 0.28159 | 1.25305 | 1.80528 | 0.29852 | 0.96282 | 0.47536 | -0.11611 | -0.12556 | -0.0701 | 0.62091 | 0.42783 |
| Csf3 | P09920 | 6.97472 | 7.2321 | 5.67858 | 8.47937 | 6.18432 | 7.06386 | 7.50984 | 6.50715 | 7.02455 | 10.05979 | 5.80507 | 9.34743 | 8.93585 | 7.80461 | 8.55931 | 8.21509 | 8.01919 | 6.7522 |
| Il33 | Q8BVZ5 | 9.55146 | 7.67801 | 6.32229 | 7.25076 | 8.0961 | 10.26081 | 8.48997 | 9.01277 | 10.1686 | 6.88181 | 8.46408 | 4.1171 | 9.3365 | 8.33124 | 9.44577 | 8.52294 | 7.49027 | 9.13361 |
| Il9 | P15247 | 0.42272 | 0.36004 | 0.53736 | 0.4554 | 0.31851 | 0.02994 | 0.41043 | 0.56051 | 1.80641 | 1.13463 | 1.1019 | 0.92764 | 0.98251 | 0.27499 | 0.20433 | 0.65967 | 0.59392 | 1.35732 |
| Cxcl2 | P10889 | 10.86036 | 10.03057 | 8.63873 | 11.8326 | 9.7319 | 10.09888 | 11.01434 | 9.90282 | 9.77571 | 12.30879 | 5.57571 | 11.00348 | 11.19285 | 10.69228 | 10.83195 | 10.3511 | 10.73674 | 9.36846 |
| Csf1 | P07141 | 5.68902 | 5.60728 | 5.31715 | 5.97331 | 5.45095 | 5.18223 | 6.14017 | 5.14556 | 5.96402 | 6.09856 | 4.69842 | 6.7065 | 5.46986 | 5.13149 | 5.67524 | 6.07064 | 5.66178 | 6.1049 |
| Ctla4 | P09793 | 9.20359 | 8.25518 | 7.23845 | 7.78619 | 8.83493 | 7.69636 | 8.47406 | 8.85422 | 10.56162 | 9.51688 | 7.25366 | 8.27304 | 7.89081 | 8.67845 | 8.59111 | 9.30295 | 8.5374 | 9.37933 |
| Ifng | P01580 | 7.6688 | 7.25372 | 5.07869 | 8.65153 | 8.23348 | 6.40698 | 10.80699 | 7.50185 | 8.9727 | 8.22975 | 6.55837 | 6.11199 | 5.9885 | 6.60879 | 5.8233 | 8.02367 | 7.77382 | 8.72068 |
| Cxcl11 | Q9JHH5 | 1.19861 | 1.41933 | 1.44993 | 1.79827 | 1.80666 | 1.41997 | 2.23895 | 1.13323 | 1.81788 | 2.41731 | 0.73815 | 1.60903 | 2.22452 | 1.35595 | 1.76739 | 2.17751 | 1.74761 | 1.42136 |
| Ccl5 | P30882 | 8.41492 | 6.88213 | 6.44402 | 7.01125 | 7.40527 | 6.44966 | 8.79263 | 7.63216 | 9.93457 | 6.83583 | 6.68704 | 5.29987 | 7.97264 | 7.19521 | 7.0376 | 8.32577 | 8.07768 | 9.31815 |
| Il17a | Q62386 | 2.1619 | 1.50536 | 2.61407 | 4.14659 | 1.51641 | 1.96391 | 1.98826 | 2.4987 | 5.76672 | 3.20911 | 0.97816 | 2.32115 | 0.84793 | 2.22852 | 1.33159 | 1.60518 | 4.82607 | 2.81266 |
| Pdcd1lg2 | Q9WUL5 | 13.85538 | 13.63578 | 12.2906 | 12.41003 | 13.70602 | 12.39053 | 14.30807 | 12.49371 | 15.23906 | 13.63811 | 12.62663 | 12.34518 | 12.10701 | 12.41788 | 12.40553 | 14.07999 | 13.89962 | 14.46557 |
| Il1b | P10749 | 9.10692 | 7.97605 | 7.32294 | 8.67452 | 8.73742 | 7.63355 | 9.47346 | 9.07015 | 10.21131 | 9.86961 | 7.09641 | 7.25223 | 8.49704 | 7.8429 | 8.26131 | 9.2066 | 9.54927 | 9.50987 |
| Il7 | P10168 | 3.19957 | 2.50358 | 2.06737 | 2.48033 | 2.87838 | 1.43848 | 3.34005 | 2.03811 | 2.84739 | 1.92764 | 2.48458 | 1.7255 | 2.56434 | 2.18413 | 1.80248 | 2.69651 | 2.46325 | 1.87943 |
| Ccl17 | Q9WUZ6 | 3.58104 | 2.85601 | 2.58112 | 1.36875 | 3.53604 | 2.47651 | 1.77816 | 2.56384 | 1.68961 | 3.52438 | 1.2208 | 1.33051 | 2.26575 | 1.29347 | 1.58324 | 3.02794 | 3.55377 | 3.25243 |
| Cd274 | Q9EP73 | 9.66967 | 8.5023 | 7.78547 | 9.1109 | 8.87942 | 9.36793 | 10.04952 | 8.45215 | 9.81298 | 9.99139 | 8.23098 | 8.96078 | 8.96408 | 8.74636 | 8.60836 | 9.2802 | 8.69313 | 9.33146 |
| Hgf | Q08048 | 10.20261 | 10.02221 | 9.14087 | 10.61194 | 9.52908 | 10.02207 | 10.28139 | 10.15185 | 11.19554 | 10.57267 | 10.21036 | 9.24691 | 10.16527 | 10.29723 | 9.88958 | 10.00084 | 10.70635 | 9.97548 |
| Il17f | Q7TNI7 | 1.38925 | 0.90004 | 0.48603 | 1.82912 | 1.66752 | 1.12329 | 1.06446 | 1.4277 | 3.2104 | 1.61178 | 1.71338 | 1.14097 | 1.21286 | 0.9491 | 1.82237 | 1.36875 | 1.96321 | 1.55769 |
| Il31 | Q6EAL8 | -0.112 | -0.14823 | -0.13383 | 0.2854 | -0.4656 | 0.48338 | 1.49709 | -0.6568 | 0.00752 | 0.41067 | -0.64558 | 0.97185 | 0.81733 | -0.10917 | 0.60119 | 0.68411 | 0.23102 | -0.45457 |
| Ccl2 | P10148 | 15.00488 | 14.81386 | 14.64987 | 15.01094 | 14.95537 | 14.79975 | 15.05548 | 14.83884 | 15.07874 | 14.98557 | 13.99794 | 14.92818 | 15.03236 | 14.82048 | 15.06795 | 15.01302 | 14.71747 | 15.00317 |
| Tnf | P06804 | 4.09264 | 3.24843 | 1.59392 | 1.77397 | 3.59381 | 2.35683 | 3.38372 | 3.41012 | 4.72445 | 4.12751 | 1.56472 | 2.66634 | 2.95111 | 3.46236 | 3.54671 | 4.20854 | 2.85864 | 3.87859 |
| Il22 | Q9JJY9 | 0.44771 | 0.39998 | 0.36382 | 0.58336 | 0.49183 | 1.00965 | 0.53227 | 0.57688 | 0.55552 | 0.62775 | 0.43794 | 0.4692 | 0.55195 | 0.47149 | 0.46097 | 0.59292 | 0.41099 | 0.61748 |
| Il10 | P18893 | 2.15749 | 1.59352 | 0.69426 | 1.62742 | 1.81768 | 2.14143 | 2.22874 | 1.52646 | 2.39922 | 1.32571 | 1.13904 | 0.82233 | 1.08668 | 1.22602 | 1.37688 | 1.43444 | 1.32172 | 1.64687 |
| Ccl12 | Q62401 | 7.89042 | 10.49436 | 10.42102 | 10.80527 | 8.60781 | 8.48133 | 9.64612 | 8.02567 | 7.79963 | 10.74352 | 7.30045 | 10.61866 | 10.16358 | 9.90541 | 10.29843 | 10.49208 | 9.84385 | 10.13109 |
| Ccl4 | P14097 | 9.96882 | 8.61152 | 7.71993 | 9.11148 | 8.5331 | 8.63132 | 9.62635 | 8.73564 | 9.7957 | 9.52267 | 7.87836 | 8.66535 | 8.72568 | 8.41195 | 8.27335 | 9.17064 | 8.77422 | 9.17988 |
| Il1a | P01582 | 8.61543 | 7.38351 | 6.20067 | 7.00811 | 7.08712 | 7.06661 | 7.6229 | 6.51711 | 7.63869 | 8.54542 | 4.51451 | 7.62793 | 7.10407 | 6.48918 | 7.13844 | 7.8316 | 7.06519 | 7.93717 |
| Ifnl2 | Q4VK74 | 1.72761 | 1.20647 | 1.2163 | 1.5168 | 1.48257 | 1.57894 | 1.17787 | 1.54536 | 1.63672 | 1.41791 | 2.1157 | 0.80817 | 1.42119 | 1.37945 | 1.4101 | 1.34808 | 1.36363 | 1.39328 |
| Il3 | P01586 | 1.66194 | 1.25995 | 0.66808 | 1.8752 | 1.90816 | 1.16438 | 2.51285 | 1.80381 | 2.32141 | 2.04409 | 0.79012 | 1.45104 | 1.59809 | 1.33156 | 1.85454 | 2.0469 | 2.06363 | 1.8593 |
| Cxcl12 | P40224 | 1.13726 | 0.60134 | 0.22378 | 1.23718 | 1.13016 | 1.16849 | 0.95205 | 0.52422 | 1.01082 | 0.96732 | 0.07906 | 0.72529 | 1.37855 | 0.85712 | 0.94827 | 1.23323 | 1.22906 | 0.58914 |
| Ccl22 | O88430 | 13.41714 | 13.09758 | 13.08077 | 13.1151 | 13.8673 | 13.21525 | 13.96299 | 13.71332 | 14.21824 | 13.63227 | 12.03912 | 13.77042 | 13.60187 | 13.31337 | 13.00665 | 13.603 | 13.1381 | 13.82712 |
| Il12a_Il12b | P43431_P43432 | 0.76641 | 0.7125 | 0.65491 | 0.87218 | 1.02173 | 1.03221 | 1.75762 | 0.71174 | 2.13591 | 1.7049 | 0.72423 | 0.83394 | 0.79972 | 1.58239 | 1.22929 | 1.63015 | 0.71254 | 0.91523 |
| Ccl11 | P48298 | 6.43898 | 5.39072 | 4.60105 | 5.38945 | 5.43813 | 7.59237 | 6.21265 | 5.97233 | 7.64691 | 5.59871 | 6.56865 | 4.17657 | 5.69038 | 7.04083 | 5.83538 | 5.7023 | 5.19662 | 6.54968 |
| Ifna2 | P01573 | 2.63913 | 2.03273 | 1.56675 | 1.97082 | 2.45303 | 0.75769 | 1.6814 | 2.05913 | 2.556 | 1.02583 | 2.43609 | 1.76134 | 1.07246 | 1.12661 | 1.17403 | 2.05572 | 1.44428 | 1.98077 |
| Csf2 | P01587 | 8.66 | 8.93594 | 7.91418 | 9.79175 | 8.85083 | 9.06654 | 9.36764 | 8.59179 | 8.42753 | 10.64251 | 5.95528 | 10.10331 | 9.8198 | 9.42001 | 9.68071 | 9.72271 | 8.75297 | 8.78848 |
| Il5 | P04401 | 3.51854 | 3.32397 | 2.14843 | 4.21319 | 3.13091 | 3.38325 | 3.9003 | 2.64263 | 3.69514 | 4.99012 | 1.6958 | 3.88008 | 3.99343 | 3.28519 | 4.00071 | 3.42257 | 3.11623 | 3.07821 |
| Cxcl9 | P18340 | 12.77184 | 12.85672 | 10.96444 | 13.50574 | 13.05324 | 10.82709 | 15.07148 | 11.879 | 14.28266 | 12.07856 | 12.16545 | 10.83999 | 10.62965 | 11.74134 | 10.38294 | 13.5455 | 13.33933 | 13.50324 |
| Il16 | O54824 | 8.74778 | 7.14294 | 6.87521 | 8.1365 | 7.00612 | 8.29197 | 7.55644 | 7.48002 | 9.27072 | 9.55136 | 8.75766 | 6.9835 | 7.83896 | 8.11675 | 7.81605 | 7.27268 | 7.84479 | 8.26884 |
| Il2 | P04351 | 3.62776 | 3.13823 | 3.00485 | 3.3917 | 4.10957 | 3.37859 | 4.09067 | 3.69045 | 5.03721 | 3.4016 | 3.03146 | 2.63913 | 3.30184 | 3.58561 | 3.72349 | 4.07966 | 3.77759 | 4.50574 |
| Il27 | Q8K3I6 | 2.82882 | 1.68707 | 2.26788 | 2.4274 | 3.05141 | 2.17986 | 3.78328 | 2.73121 | 3.61472 | 3.82347 | 2.01442 | 2.24004 | 2.97491 | 2.6237 | 2.73449 | 3.51632 | 3.21101 | 3.22301 |

Olink, Olink Proteomics; Ctrl, control; UHDR-RT, ultra-high dose rate radiotherapy; Conv-RT, conventional radiotherapy.
